# Supplementary material for: Designed peptides as nanomolar cross-amyloid inhibitors acting via supramolecular nanofiber co-assembly
Source: Nat Commun. 2022 Aug 25;13:5004. doi: 10.1038/s41467-022-32688-0 (PMC9411207; doi:10.1038/s41467-022-32688-0)
Supplement: Supplementary file 1 — Supplementary Information [file 41467_2022_32688_MOESM1_ESM.pdf]

# Supplementary Information

## Designed peptides as nanomolar cross-amyloid inhibitors acting via supramolecular nanofiber co-assembly

Karin Taş[1], Beatrice Dalla Volta[1], Christina Lindner[1#], Omar El Bounkari[2], Kathleen Hille[1], Yuan Tian[2], Xènia Puig-Bosch[3], Markus Ballmann[3], Simon Hornung[1], Martin Ortner[1$], Sophia Prem[1§], Laura Meier[4], Gerhard Rammes[3], Martin Haslbeck[4], Christian Weber[5,6,7,8], Remco T.A. Megens[5,7,9], Jürgen Bernhagen[2,6], Aphrodite Kapurniotu[1*]

[1]Division of Peptide Biochemistry, TUM School of Life Sciences, Technical University of Munich (TUM), 85354 Freising, Germany; [2]Division of Vascular Biology, Institute for Stroke and Dementia Research (ISD), Klinikum der Universität München, Ludwig-Maximilian-University (LMU), 81377 Munich, Germany; [3]Department of Anesthesiology and Intensive Care, Technical University of Munich/Klinikum Rechts der Isar, 81675 München, Germany; [4]Center for Protein Assemblies, Department of Chemistry, Technical University of Munich, 85748 Garching, Germany; [5]Institute for Cardiovascular Prevention, Klinikum der Universität München, Ludwig-Maximilian-University Munich (LMU), 80336 Munich, Germany; [6]Munich Cluster for Systems Neurology (SyNergy), 81377 Munich, Germany; [7]German Centre for Cardiovascular Research (DZHK), partner site Munich Heart Alliance, 80802 Munich, Germany; [8]Department of Biochemistry, Cardiovascular Research Institute Maastricht (CARIM), Maastricht University, 6229 Maastricht, The Netherlands; [9]Department of Biomedical Engineering, Cardiovascular Research Institute Maastricht (CARIM), Maastricht University, 6229 Maastricht, The Netherlands

*Correspondence:
Professor Aphrodite Kapurniotu, PhD
E-mail: akapurniotu@mytum.de

**Table of Contents**

- Supplementary Tables 1-4, pp. 2-4
- Supplementary Fig. 1-27 (incl. legends), pp. 5-35
- References included in Supplementary Information, pp. 36

## Supplementary Tables

**Supplementary Table 1.** Amino acid sequences and abbreviations of template segment Aβ(15-40) (light blue background) and synthesized and tested peptides i.e. the 6 amyloid inhibitors (ACMs; grey background) and the non-inhibitors. The sequence of segment Aβ(24-26) (highlighted in yellow) and applied loop tripeptide segments (LTS) are shown by the three-letter code; different colors are used for the different LTS; peptides are arranged in groups according to their LTS. N-methylated amino acids are shown in bold. All peptides have a free N-terminal amino group ($NH_2$-) and are C-terminal carboxylic acids (-COOH).

| Peptide Sequence | Abbreviation |
|---|---|
| QKLVFFAED – Val-Gly-Ser – NKGAIIGLMVGGVV | Aβ(15-40) |
| QKLVFFAED – Nle-Nle-Nle – NKGAIIGLNleVGGVV | Nle3 |
| QKL**(N-Me)V**F**(N-Me)F**AED – Nle-Nle-Nle – NKGAIIGLNleVGGVV | Nle3-VF |
| QK**(N-Me)L**V**(N-Me)F**FAED – Nle-Nle-Nle – NKGAIIGLNleVGGVV | Nle3-LF |
| QKLVFFAED – Nle-Nle-Nle – NK**(N-Me)G**A**(N-Me)I**IGLNleVGGVV | Nle3-GI |
| QKLVFFAED – Nle-Nle-Nle – NK**(N-Me)G**AII**(N-Me)G**LNle VGGVV | Nle3-GG |
| QKLVFFAED – Leu-Leu-Leu – NKGAIIGLNleVGGVV | L3 |
| QKL**(N-Me)V**F**(N-Me)F**AED – Leu-Leu-Leu – NKGAIIGLNleVGGVV | L3-VF |
| QK**(N-Me)L**V**(N-Me)F**FAED – Leu-Leu-Leu – NKGAIIGLNleVGGVV | L3-LF |
| QKL**(N-Me)V**F**(N-Me)F**AED – Phe-Phe-Phe – NKGAIIGLNleVGGVV | F3-VF |
| QK**(N-Me)L**V**(N-Me)F**FAED – Phe-Phe-Phe – NKGAIIGLNleVGGVV | F3-LF |
| QKLVFFAED – Val-Gly-Ser – NKGAIIGLNleVGGVV | VGS (Aβ(15-40)) |
| QKL**(N-Me)V**F**(N-Me)F**AED – Val-Gly-Ser – NKGAIIGLNleVGGVV | VGS-VF |
| QK**(N-Me)L**V**(N-Me)F**FAED – Val-Gly-Ser – NKGAIIGLNleVGGVV | VGS-LF |
| QKLVFFAED – Arg-ArgArg – NKGAIIGLNleVGGVV | R3 |
| QKL**(N-Me)V**F**(N-Me)F**AED – Arg-Arg-Arg – NKGAIIGLNleVGGVV | R3-VF |
| QK**(N-Me)L**V**(N-Me)F**FAED – Arg-Arg-Arg – NKGAIIGLNleVGGVV | R3-LF |
| QKLVFFAED – Gly-Gly-Gly – NKGAIIGLNleVGGVV | G3 |
| QKL**(N-Me)V**F**(N-Me)F**AED – Gly-Gly-Gly – NKGAIIGLNleVGGVV | G3-VF |

**Supplementary Table 2.** Widths of fIAPP and fibrils found in aged IAPP, IAPP/ACM and IAPP/VGS-VF mixtures as determined by TEM.

| Peptide or peptide mixture | Fibril width (nm)[a] |
|---|---|
| **IAPP** | 9.7 (±2.3) |
| **IAPP + Nle3-VF** | 8.3 (±1.9) |
| **IAPP + L3-VF** | 7.7 (±1.5) |
| **IAPP + F3-VF** | 8.7 (±1.8) |
| **IAPP + Nle3-LF** | 6.1 (±1.1) |
| **IAPP + L3-LF** | 7.9 (±1.8) |
| **IAPP + F3-LF** | 6.6 (±1.2) |
| **IAPP + VGS-VF** | 8.5 (±2.0) |

[a] Measured in 7 day-aged IAPP and IAPP/peptide mixtures (from Fig. 1d,f; TEM images Fig. 2f). Data are means (±SD) from 21-47 fibrils as specified below: n=42 (IAPP), 47 (IAPP + Nle3-VF), 25 (IAPP + L3-VF), 21 (IAPP + F3-VF), 22 (IAPP + Nle3-LF) and (IAPP + F3-LF), 24 (IAPP + L3-LF), and 30 (IAPP + VGS-VF). Measured in grids from 3 (IAPP + Nle3-VF) or 2 (IAPP and IAPP + VGS-VF) biologically independent samples and from 1 sample of each of the other mixtures.

**Supplementary Table 3.** Lengths and widths of fAβ42 and fibrils found in Aβ42/ACM mixtures as determined by TEM.

| Peptide or peptide mixture | Fibril length (nm)[a] | Fibril width (nm)[a] |
|---|---|---|
| **Aβ42** | 154 (±58) | 7.8 (±1.6) |
| **Aβ42 + Nle3-VF** | 472 (±138) | 7.3 (±1.7) |
| **Aβ42 + Nle3-LF** | 590 (±268) | 7.5 (±1.1) |
| **Aβ42 + L3-VF** | 458 (±106) | 6.9 (±1.4) |
| **Aβ42 + L3-LF** | 347 (±72) | 7.0 (±2.2) |
| **Aβ42 + F3-VF** | 354 (±117) | 6.9 (±1.3) |
| **Aβ42 + F3-LF** | 358 (±118) | 7.3 (±1.5) |

[a] Measured in 6 day-aged solutions of Aβ42 and its mixtures (1/1) with ACMs (from Fig. 6b; TEM images Fig. 6d). Data are means (±SD) from 15-23 (lengths) or 20-23 (widths) fibrils (1 grid per sample) as specified below (see also bar diagram in Fig. 6d): Lengths: n=22 (Aβ42 and Aβ42 + F3-LF), 20 (Aβ42 + Nle3-VF, Aβ42 + Nle3-LF, and Aβ42 + L3-LF), 15 (Aβ42 + L3-VF), and 23 (Aβ42 + F3-VF). Widths: n=21 (Aβ42 and Aβ42 + Nle3-VF), 20 (Aβ42 + Nle3-LF and Aβ42 + L3-LF), 23 (Aβ42 + L3-VF and Aβ42 + F3-LF), and 22 (Aβ42 + F3-VF).

**Supplementary Table 4.** Molecular weights (M) of synthesized peptides which were used in this study as determined by MALDI-TOF-MS or ESI-MS (*).

| Peptide | $[M+H]^+$ ($[M+Na]^+$) calculated (g/mol) | $[M+H]^{+[a]}$ or $[M+Na]^{+[b]}$ found (g/mol) |
|---|---|---|
| Nle3 | 2726.61 (2748.59) | 2748.71[b] |
| Nle3-VF | 2754.67 (2776.65) | 2776.89[b] |
| Nle3-LF | 2754.67 (2776.65) | 2776.83[b] |
| Nle3-GI | 2754.67 (2776.65) | 2776.50[b] |
| Nle3-GG | 2754.67 (2776.65) | 2777.13[b] |
| L3 | 2726.61 (2748.59) | 2727.00[a]* |
| L3-VF | 2754.67 (2776.65) | 2776.99[b] |
| L3-LF | 2754.67 (2776.65) | 2757.00 [a]* |
| F3-VF | 2856.63 (2878.61) | 2878.92[b] |
| F3-LF | 2856.63 (2878.61) | 2878.73[b] |
| VGS | 2630.48 (2652.46) | 2652.35[b] |
| VGS-VF | 2658.54 (2680.52) | 2681.37[b] |
| VGS-LF | 2658.54 (2680.52) | 2680.47[b] |
| R3 | 2855.67 (2877.65) | 2855.97[a] |
| R3-VF | 2883.73 (2905.71) | 2884.80[a]* |
| R3-LF | 2883.73 (2905.71) | 2883.99[a] |
| G3 | 2558.43 (2580.41) | 2580.42[b] |
| G3-VF | 2586.49 (2608.47) | 2609.00[b] |
| Nle3-VF(15-23)[c] | 1124.62 (1146.60) | 1146.62[b] |
| Nle3-VF(27-40)[c] | 1309.81 (1331.79) | 1331.93[b] |
| Nle3-VF(21-40)[c] | 1964.17 (1986.15) | 1986.21[b] |
| Fluos-Nle3-VF | 3112.97 (3134.95) | 3134.93[b] |
| Fluos-Nle3-LF | 3112.97 (3134.95) | 3135.16[b] |
| Fluos-L3-VF | 3112.97 (3134.95) | 3134.94[b] |
| Fluos-L3-LF | 3112.97 (3134.95) | 3134.75[b] |
| Fluos-F3-VF | 3214.93 (3236.91) | 3237.05[b] |
| Fluos-F3-LF | 3214.93 (3236.91) | 3236.59[b] |
| Fluos-VGS-VF | 3016.84 (3038.82) | 3038.84[b] |
| Aβ42 | 4512.28 (4534.26) | 4512.83[a]* |
| rat IAPP[1] | 3918.96 (3940.94) | 3923.54[a] |
| IAPP-GI[2] | 3929.92 (3951.90) | 3930.47[a] |
| IAPP[2] | 3901.86 (3923.84) | 3902.41[a] |
| Biotin-IAPP[2] | 4240.19 (4262.17) | 4241.13[a] |
| TAMRA-IAPP | 4314.29 (4336.27) | 4313.95[a] |
| Fluos-IAPP[2] | 4260.16 (4282.14) | 4260.48[a] |
| Atto647N-Nle3-VF | 3382.57 (3404.55) | 3381.85[a] |

M, monoisotopic mass; [a] $[M+H]^+$; [b] $[M+Na]^+$; [c] numbering of residues/segments of partial Aβ(15-40) or ACM analogs according to their sequence numbers in Aβ40.

# Supplementary Figures 1-27

Supplementary Fig. 1

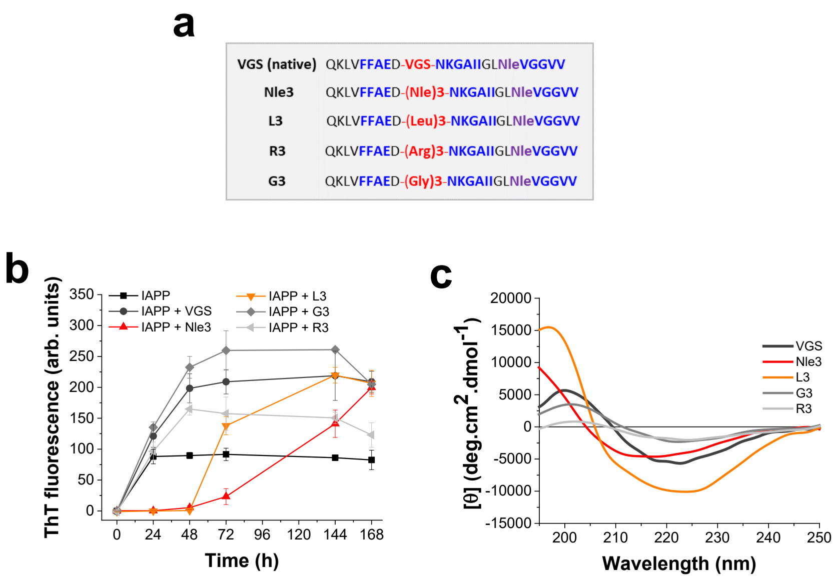


**Supplementary Fig. 1.** Identification of best-suited LTS for inhibitor design. **a** Sequences of Aβ(15-40) (abbreviated "VGS") and designed analogs thereof (red: LTS; blue: "hot segments"[3]; purple: Met35Nle substitution). **b** Effects of VGS and analogs on IAPP fibril formation: fibrillogenesis of IAPP (16.5 µM) with or without the peptides determined by the ThT binding assay (IAPP/peptide 1/2) (means ± SD, 3 independent assays). **c** Far-UV CD spectra of VGS and its analogs (5 µM, pH 7.4). Consistent results were obtained in 2 independent experiments.

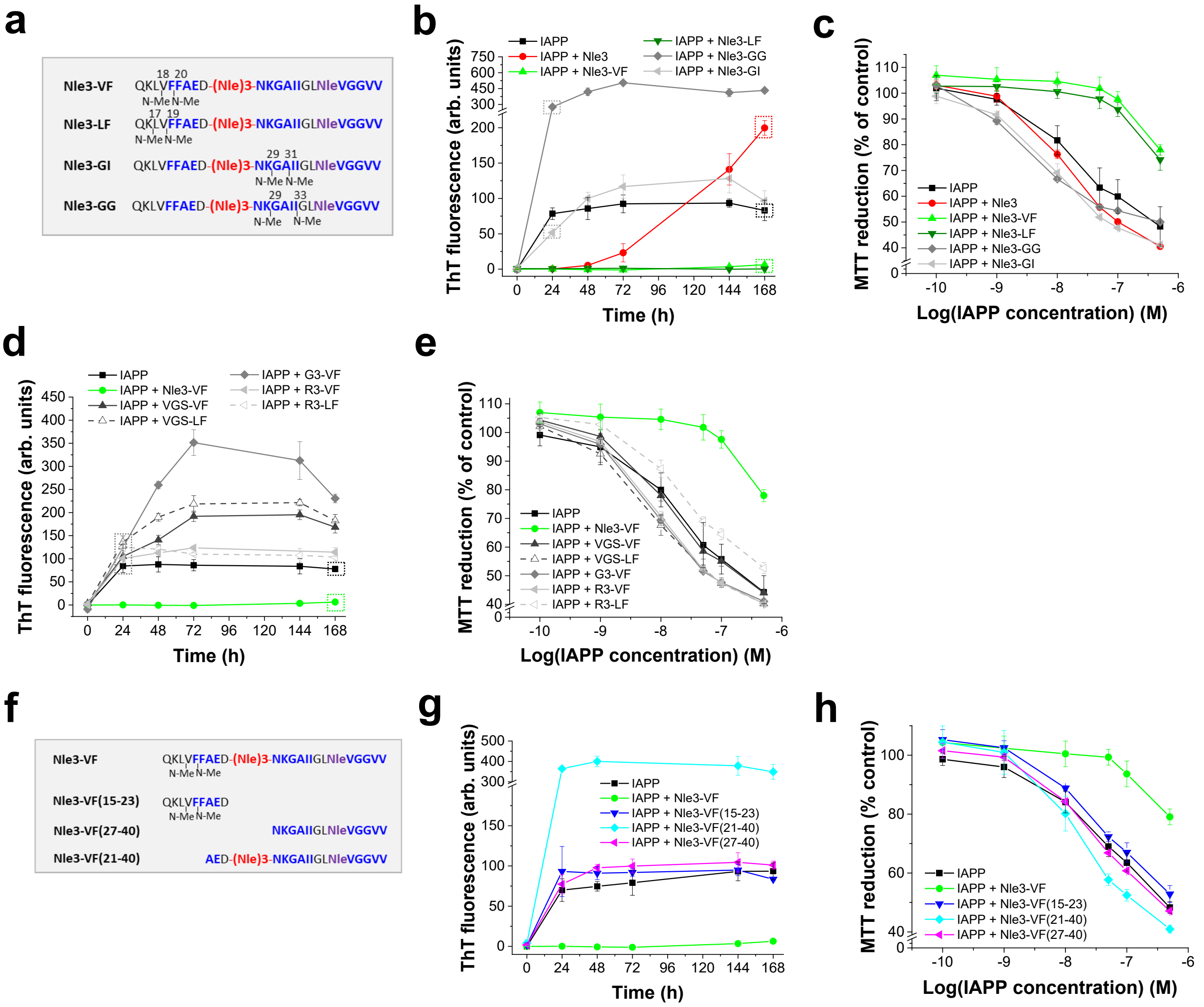


**Supplementary Fig. 2.** Identification of best-suited sequence positions for N-methylations (**a**-**e**) and effects of partial segments of Nle3-VF on IAPP amyloid self-assembly (**f**-**g**). **a** Sequences of the four designed N-methylated Nle3 analogs (red: (Nle)3; blue: hot segments[3]; purple: Met35Nle substitution); numbers indicate positions of N-methylated residues (for numbering the residues of all analogs, their numbers in the Aβ40 sequence were used). **b** Effects of the Nle3 analogs on IAPP fibrillogenesis. Fibrillogenesis of IAPP alone (16.5 μM) or its mixtures with the different analogs was studied via the ThT binding assay (IAPP/peptide 1/2). Dashed boxes indicate incubation time points at which parts of the solutions were used for the MTT reduction assay shown in **c**. Of note, data of Nle3-VF and Nle3-LF are also shown in Fig. 1d,f. Data are means ± SD from 3 independent assays. **c** Effects of Nle3 analogs on formation of cytotoxic IAPP assemblies. Solutions of **b** (24 h or 7 days aged as indicated) were added to RIN5fm cells; cell damage was determined via MTT reduction (means ± SD, 3 independent assays, n=3 technical replicates each). Of note, data of Nle3-VF and Nle3-LF is also shown in Fig. 1e,g. **d** Effects of Val18Phe20- (-VF) or Leu17Phe19- (-LF) N-methylated analogs of the non-inhibitors VGS, R3, and G3 on IAPP fibrillogenesis as compared to inhibitor Nle3-VF. Fibrillogenesis of IAPP alone (16.5 μM) or its mixtures with the analogs was studied via ThT binding (IAPP/peptide 1/2). Dashed boxes indicate incubation time points at which parts of the solutions were used for the MTT reduction assay shown in **e**. Of note, data of Nle3-VF are also shown in Fig. 1d. Data are means ± SD from 3 independent assays. **e** Effects of peptides studied under **d** on IAPP cytotoxicity. Solutions of **d** were added to RIN5fm cells; cell damage determined via MTT reduction (means ± SD, 3 independent assays, n=3 technical replicates each). Of note, data of Nle3-VF are also shown in Fig. 1e. **f** Sequences and abbreviations of synthesized and tested partial segments of Nle3-VF (see **g**,**h**) (color code: same as in **a**). **g** Effects of the partial segments of Nle3-VF shown in **f** on IAPP fibrillogenesis as compared to Nle3-VF. Fibrillogenesis of IAPP alone (16.5 μM) or its mixtures with each of the segments was studied via ThT binding (IAPP/segment 1/2). Data are means ± SD from 3 independent assays. **h** Effects of peptides studied under **g** on IAPP cytotoxicity. Solutions of **g** (at 24 h) were added to RIN5fm cells; cell damage determined via MTT reduction (means ± SD, 3 independent assays, n=3 technical replicates each).

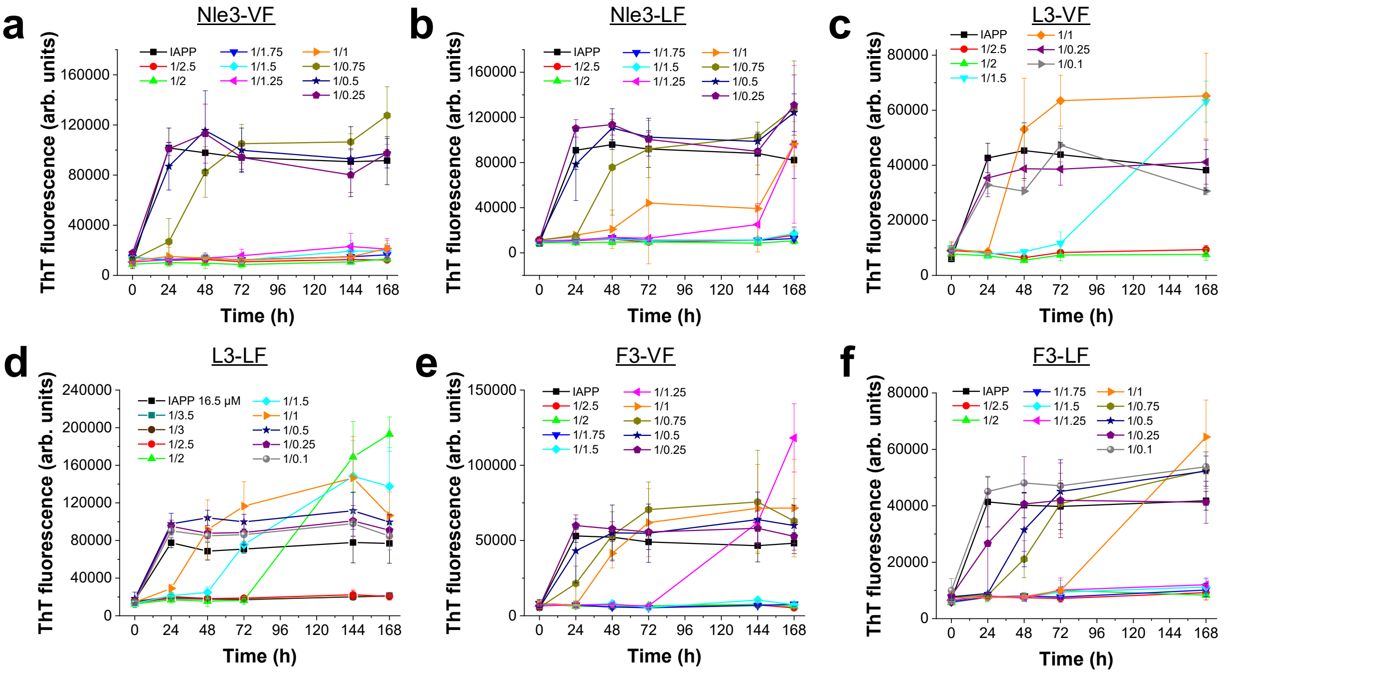


**Supplementary Fig. 3.** Concentration-dependence of inhibitory effects of ACMs on IAPP amyloid self-assembly. Fibrillogenesis of IAPP (16.5 μM) alone or in presence of Nle3-VF (**a**), Nle3-LF (**b**), L3-VF (**c**), L3-LF (**d**), F3-VF (**e**), and F3-LF (**f**) at the indicated IAPP/ACM ratios was followed by the ThT binding assay. Data are means ± SD from 3-8 independent assays as specified here: Fig. 3a: n=8 (IAPP/Nle3-VF 1/2), n=5 (IAPP), n=4 (IAPP/Nle3-VF 1/1), and n=3 all other IAPP/Nle3-VF ratios; Fig. 3b: n=8 (IAPP/Nle3-LF 1/2), n=6 (IAPP), n=4 (IAPP/Nle3-LF 1/1.75 & 1/1.5), and n=3 all other IAPP/Nle3-LF ratios; Fig. 3c-f: n=3 for all IAPP/ACM ratios.

Supplementary Fig. 4

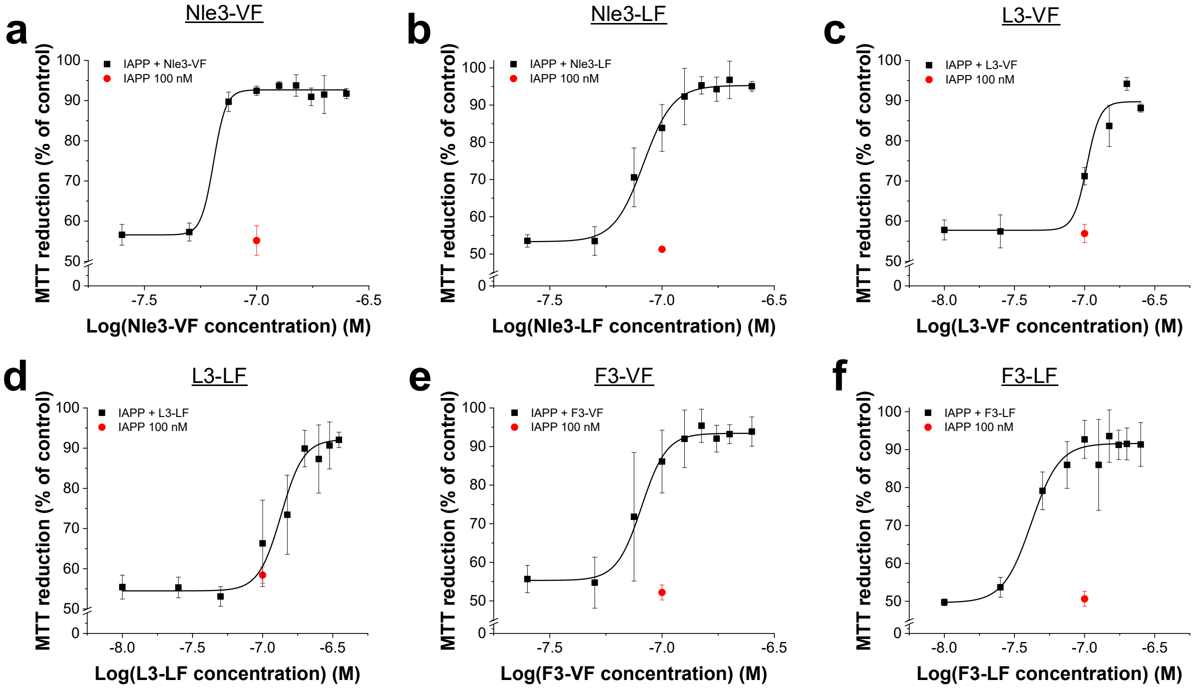


**Supplementary Fig. 4.** Determination of $IC_{50}$ values of inhibitory effects of ACMs on formation of cell-damaging IAPP assemblies. Aged solutions (24 h) of IAPP alone (100 nM) or its mixtures with different amounts of ACMs were added to RIN5fm cells. Cell damage was determined by the MTT reduction assay for mixtures of IAPP with Nle3-VF (**a**), Nle3-LF (**b**), L3-VF (**c**), L3-LF (**d**), F3-VF (**e**) and F3-LF (**f**) as indicated; cytotoxicity of IAPP alone is included in each graph for comparison (red symbol). Data and determined $IC_{50}$ values are means ± SD from 3 independent assays (n=3 technical replicates each).

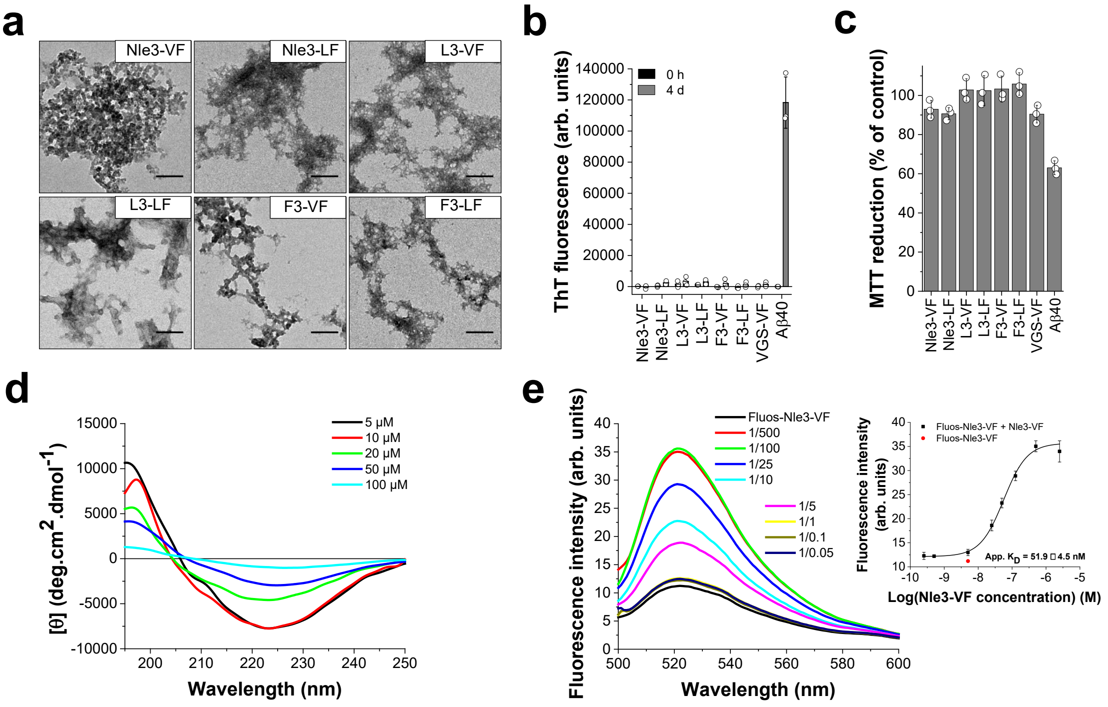


**Supplementary Fig. 5.** ACMs self-assemble into soluble, non-fibrillar, β-sheet rich, and non-toxic aggregates. **a** TEM examination of aged solutions of the six ACMs (100 μM; 4 days) reveals amorphous aggregates as main species; scale bars: 100 nm. Consistent results found for Nle3-VF, Nle3-LF, and L3-LF in 3 and for L3-VF, F3-VF, and F3-LF in 2 similar independent experiments. **b** ACM aggregates do not bind ThT. ThT binding properties of the aged solutions of ACMs used in **a** and the non-inhibitor VGS-VF are shown (100 μM; 4 days). The ThT binding properties of an aged Aβ40 solution (100 μM; 4 days) is shown for comparison. Data are means ± SD from 3 independent assays. **c** ACM aggregates are not cytotoxic. Effects of aged solutions of ACMs and VGS-VF (4 days aged solutions from **a**; at 20 μM) on PC12 cell viability as determined via the MTT reduction assay. For comparison, effects of an aged fibrillar Aβ40 (solution from **b**; at 20 μM) are shown. Data are means ± SD from 3 independent assays, n=3 technical replicates each. **d** Nle3-VF oligomerization studied by far-UV CD spectroscopy. CD spectra at different peptide concentrations as indicated (aq. solution, pH 7.4) are shown. Loss of signal was indicative of oligomerization; however, no turbidity or precipitation was observed. Results were consistent with results obtained with other ACMs. **e** Self-assembly of Nle3-VF studied by fluorescence spectroscopic titrations. Emission spectra of Fluos-Nle3-VF (5 nM) alone and with various Nle3-VF amounts as indicated (Fluos-Nle3-VF/Nle3-VF) (pH 7.4); spectra are from one representative assay out of three. Inset, binding curve (data means ± SD from 3 independent binding curves); determined app. $K_D$ = 51.9 ± 4.5 nM (mean ± SD from 3 independent binding curves). The value of Fluos-IAPP alone is also shown for comparison.

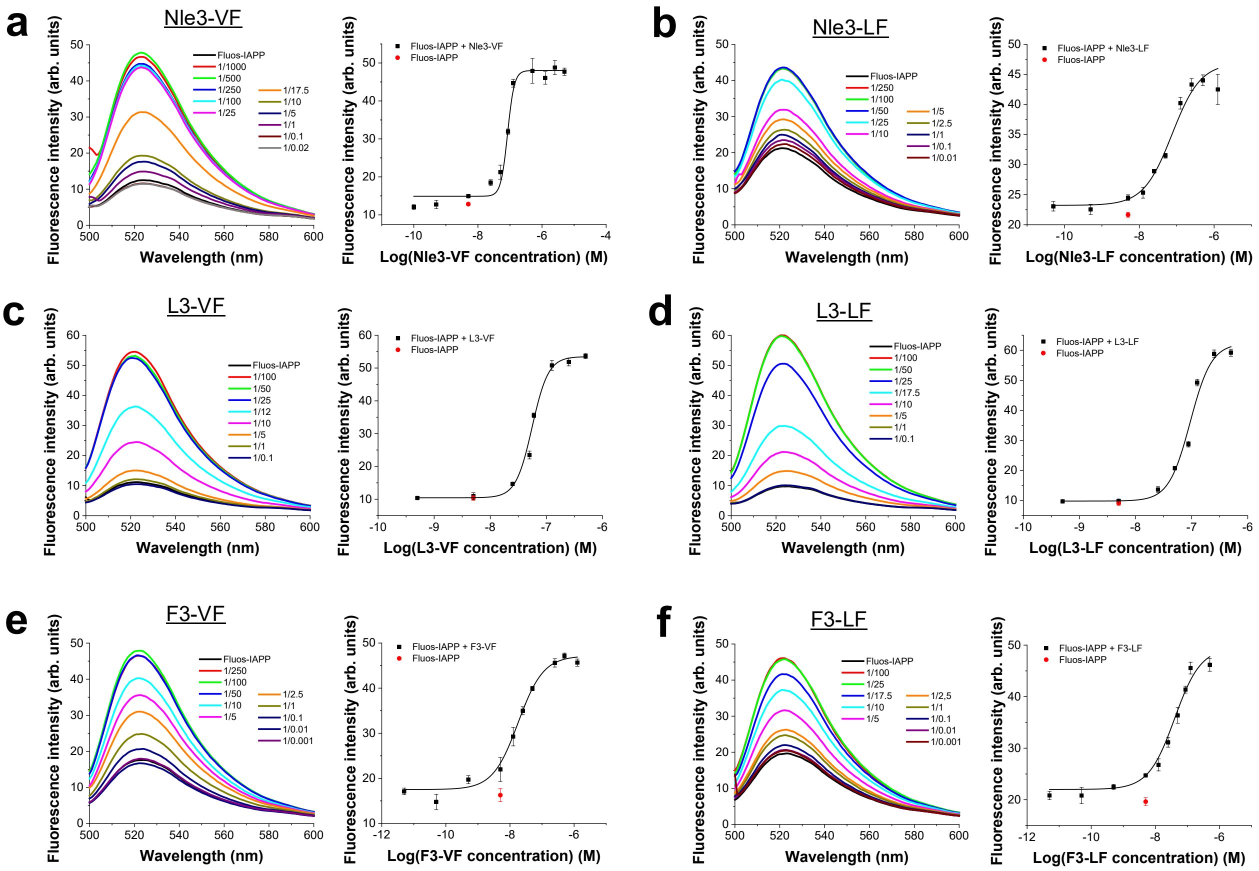


**Supplementary Fig. 6.** Determination of binding affinities of IAPP/ACM interactions by fluorescence spectroscopic titrations. Fluorescence emission spectra of Fluos-IAPP (5 nM) alone or in the presence of different molar ratios of ACMs as indicated (Fluos-IAPP/ACM) (pH 7.4) are shown on the left side of each figure panel; spectra are from one representative assay out of three independent assays. Data on the interactions of Fluos-IAPP with (**a**) Nle3-VF, (**b**) Nle3-LF, (**c**) L3-VF, (**d**) L3-LF, (**e**) F3-VF, and (**f**) F3-LF are shown as indicated. On the right side of each figure panel, the corresponding binding curves are shown; data are means ± SD from 3 independent binding curves. The value of Fluos-IAPP alone is also shown for comparison. Determined app. $K_D$s (means ± SD from 3 independent binding curves) are in Table 1.

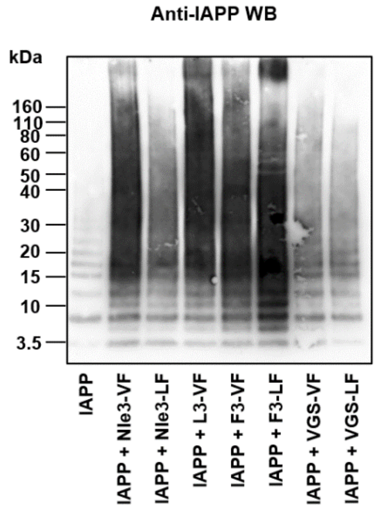


**Supplementary Fig. 7.** IAPP/ACM interactions yield hetero-di-/-tri-/-tetramers and large amounts of poorly resolved medium-to-high MW hetero-assemblies. Characterization of hetero-complexes in aged mixtures of IAPP with 5 ACMs (IAPP/peptide, 1/2; IAPP, 30 μM; pH 7.4; 7 days) via cross-linking, NuPAGE, and Western blot with anti-IAPP antibody. For comparison, assemblies present in aged IAPP (30 μM; pH 7.4; 7 days) and its mixtures with non-inhibitors VGS-VF and VGS-LF (IAPP/peptide, 1/2; IAPP, 30 μM; pH 7.4; 7 days) are also shown. Data are representative from 18 (IAPP), 13 (IAPP + Nle3-VF), 7 (IAPP + VGS-VF), and 2 (all other mixtures) membranes.

Supplementary Fig. 8a

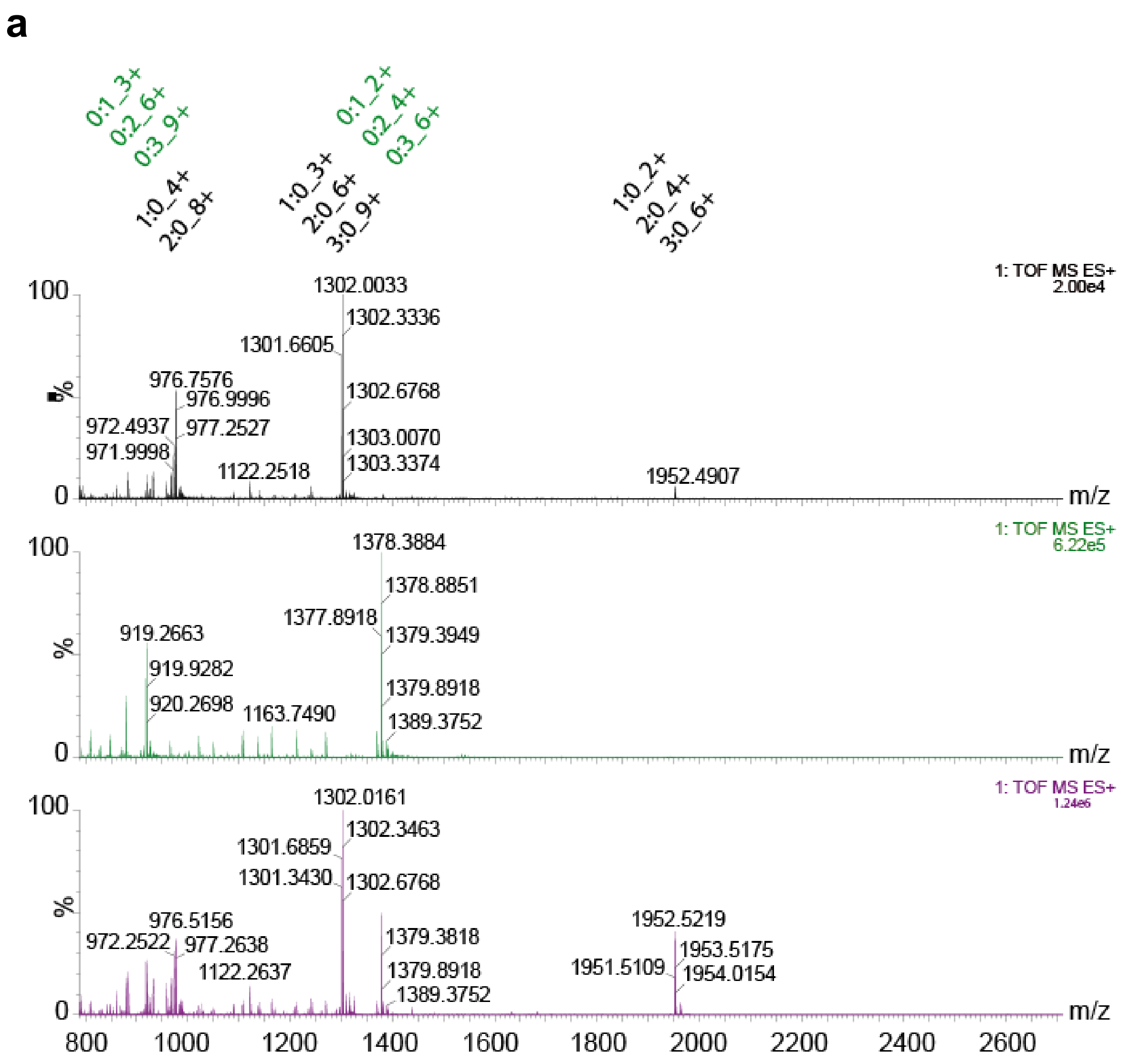

**b**

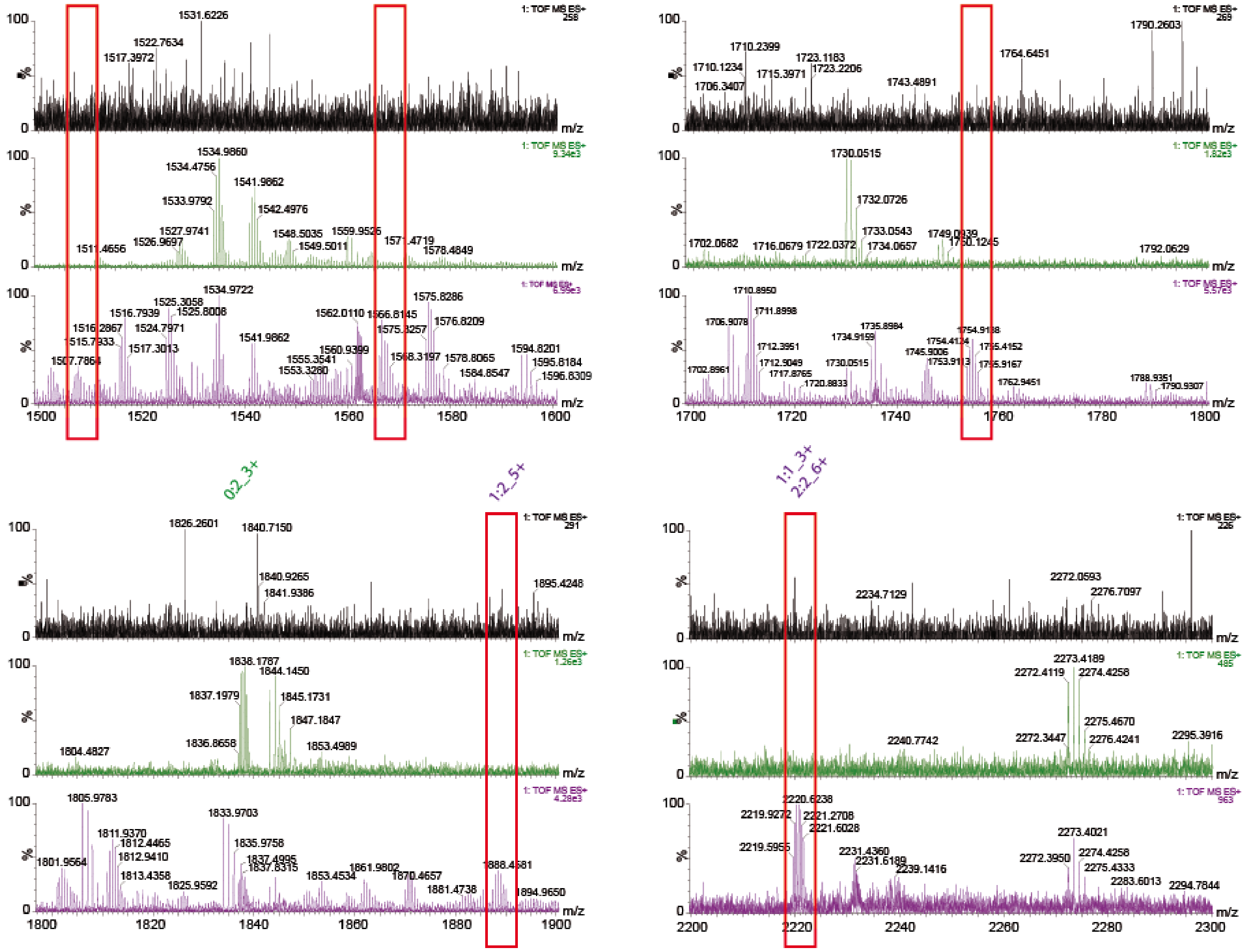


**Supplementary Fig. 8.** Identification of homo- or hetero-oligomers present in freshly made solutions of IAPP alone, Nle3-VF alone, and IAPP/Nle3-VF (1/2) by ESI-IMS-MS. **a** Comparison of ESI-MS mass spectra of IAPP alone (black), Nle3-VF alone (green) and IAPP/Nle3-VF (pink) (IAPP 16.5 μM, Nle3-VF 30 μM, pH 7.4; 0 h). Additional numbers above peaks (above the y-axis height) denote the oligomer order (IAPP: mono-/di-/trimers; Nle3-VF: mono-/di-/trimers) and the respective positive charge state of the ion-series. Coloring denotes the spectrum depicting the corresponding ion-series. **b** A selection of zoom graphs of the ESI-MS mass spectra which indicated IAPP/Nle3-VF hetero-dimers, -trimers and -tetramers (highlighted in red boxes). Additional numbers above peaks (above the y-axis height) denote the oligomer order and the respective positive charge state of the ion-series. Coloring denotes the spectrum depicting the corresponding ion-series. Data are representative of 2 experiments.

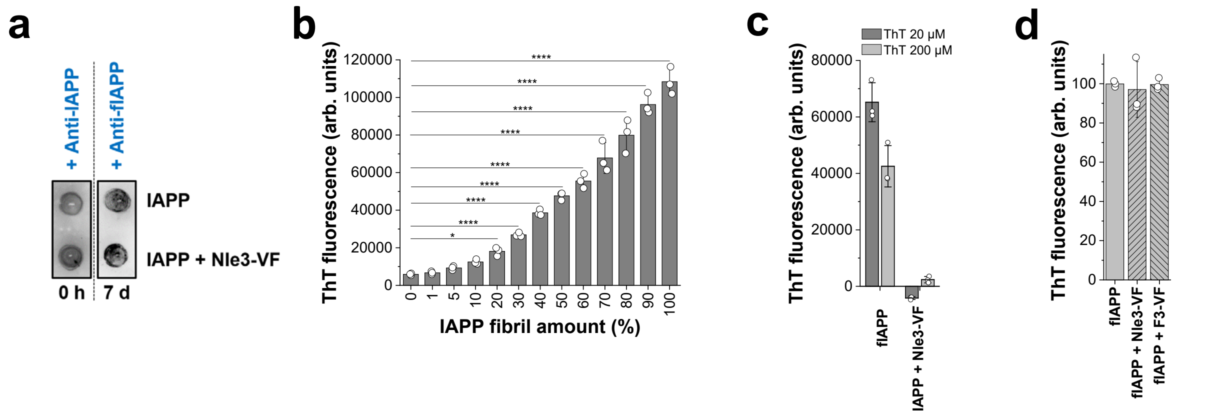


**Supplementary Fig. 9.** Evidence that ThT-invisible fibrils found in IAPP/ACM mixture are neither fIAPP rests which were not detected by ThT nor fIAPP with non-specifically bound ACM. **a** Dot blot analysis shows that same amounts of IAPP were present in aliquots of IAPP alone or IAPP/Nle3-VF mixtures used for ThT binding, MTT reduction assays, and TEM. These results excluded the possibility that the lack of ThT binding of the fibrils found in aged IAPP/Nle3-VF mixtures might be due to the presence of less amounts of fIAPP in the aliquots of the mixtures than in the aged IAPP alone solutions, e.g. caused by fIAPP sticking to microtube walls in the presence of Nle3-VF. Aliquots (equal volumes) from freshly made (0 h) or 7 day-aged solutions of IAPP (fIAPP; 16.5 µM) or IAPP/Nle3-VF (1/2) mixtures were spotted onto nitrocellulose membrane. IAPP (1.3 µg) in freshly made (0 h) solutions was quantified with an anti-IAPP antibody whereas in 7 day-aged solutions (consisting mostly of fibrils) by a fibril-specific anti-fIAPP antibody[4]. Results on 7 day-aged solutions are representative of 2 independent dot blots; consistent findings also in Fig. 4g. **b** The applied ThT assay had a high fIAPP detection sensitivity. Aged IAPP (16.5 µM; 7 days) consisting mostly of fIAPP (based on TEM and ThT binding (Fig. 1d & 2f) was serially diluted as indicated and fibrils were quantified by the ThT binding assay. ThT signals that differed significantly from the buffer were found for fIAPP concentrations ≥3.3 µM corresponding to 20% of total fIAPP amount. Data are means ± SD (3 independent assays); ****P<0.0001 for 100%-30% fIAPP versus buffer and *P<0.05 (P value 0.0321) for 20% fIAPP versus buffer (one-way ANOVA & Bonferroni) as indicated. **c** Lack of ThT reactivity of fibrils in IAPP/Nle3-VF mixtures is not due to competition between ThT and Nle3-VF for binding to fIAPP. ThT binding of fIAPP (16.5 µM; 96 h aged) and an IAPP/Nle3-VF (1/2) mixture (96 h-aged) was determined using 20 and 200 µM ThT and no differences were observed (data are means ± SD from 3 independent assays; buffer values were subtracted). **d** fIAPP does not lose its ThT binding potential after co-incubation ("coating") with ACMs. fIAPP (IAPP 16.5 µM, 9 day-aged) before and 1 day after co-incubation with Nle3-VF or F3-VF (33 µM). Data are means ± SD from 3 independent assays; see also related assay in Supplementary Fig. S13b.

Supplementary Fig. 10

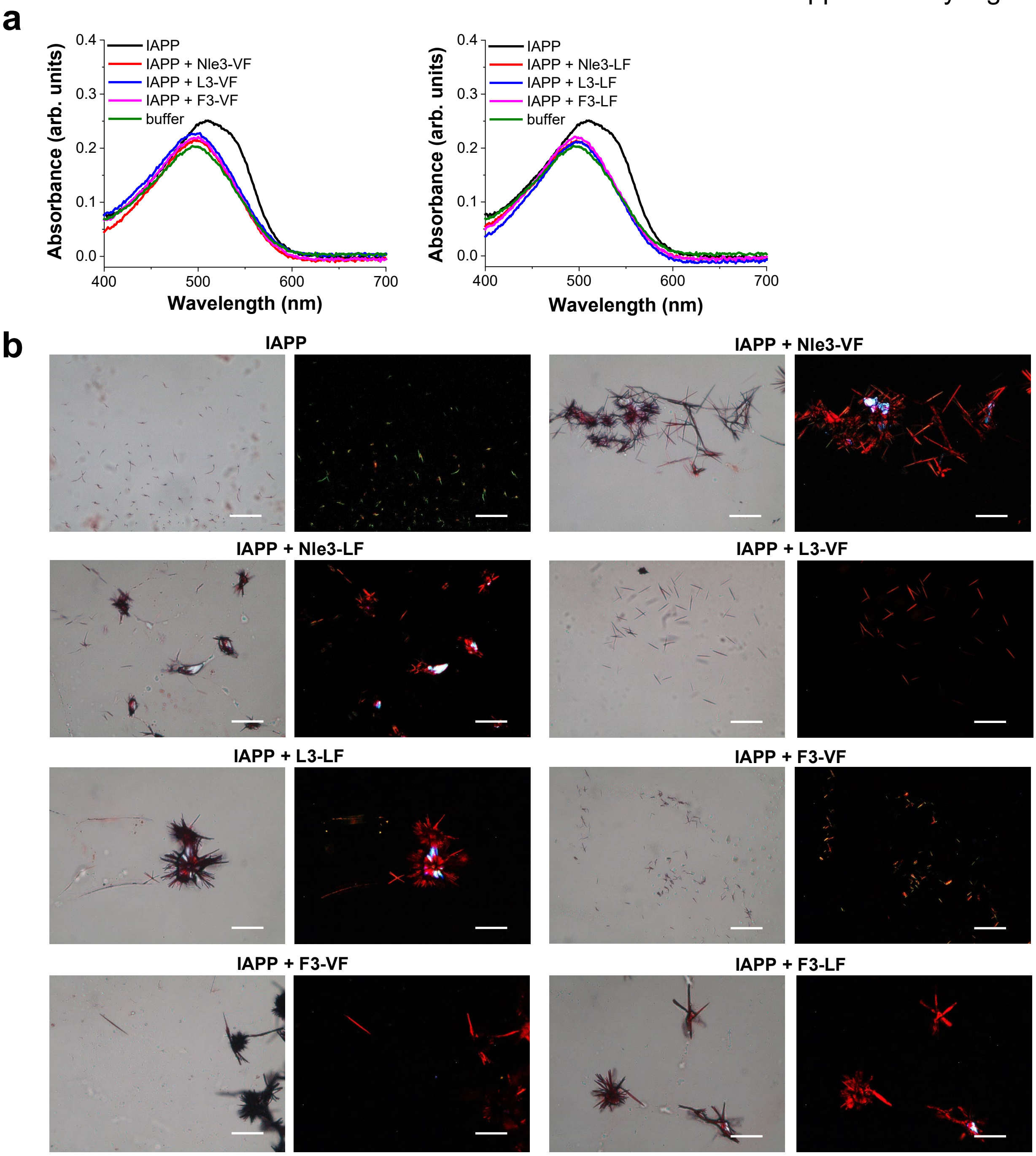


**Supplementary Fig. 10. ThT-invisible fibrillar assemblies of IAPP/ACM mixtures do not bind CR and are not birefringent in polarized light upon staining with CR. a** CR spectral shift assay[5]: No spectral shift of CR in the presence of the fibrils of the IAPP/ACM mixtures is observed whereas a clear shift is found for fIAPP. Representative absorption spectra of CR in the presence of fIAPP (16.5 µM, 7 days aged IAPP), various different IAPP/ACM mixtures (1/2; 7 days aged) consisting mostly of fibrils (see Fig. 2f), and buffer alone (control). Left side: IAPP mixtures with Nle3-VF, L3-VF, and F3-VF; right side: IAPP mixtures with Nle3-LF, L3-LF, and F3-LF. Data are representative from 4 (fIAPP), 3 (IAPP/Nle3-VF), 2 (IAPP/L3-VF and IAPP/F3-VF), and 1 (IAPP/Nle3-LF, IAPP/L3-LF, and IAPP/F3-LF) independent assay(s). **b** CR staining of fIAPP and fibrils in IAPP/ACM mixtures (solutions of **a**): Microscopic images from examinations under bright field or between cross-polarizer/analyzer reveal that in contrast to fIAPP most of the fibrillar assemblies in IAPP/ACM solutions did not exhibit the green/yellow birefringence under polarized light typical for amyloid. Only in aged IAPP/F3-VF, some green/yellow birefringent fibrillar assemblies were also observed. Scale bars, 10 µm. Images are representative from 3 or 1 biologically independent fIAPP or IAPP/ACM mixture sample(s), respectively.

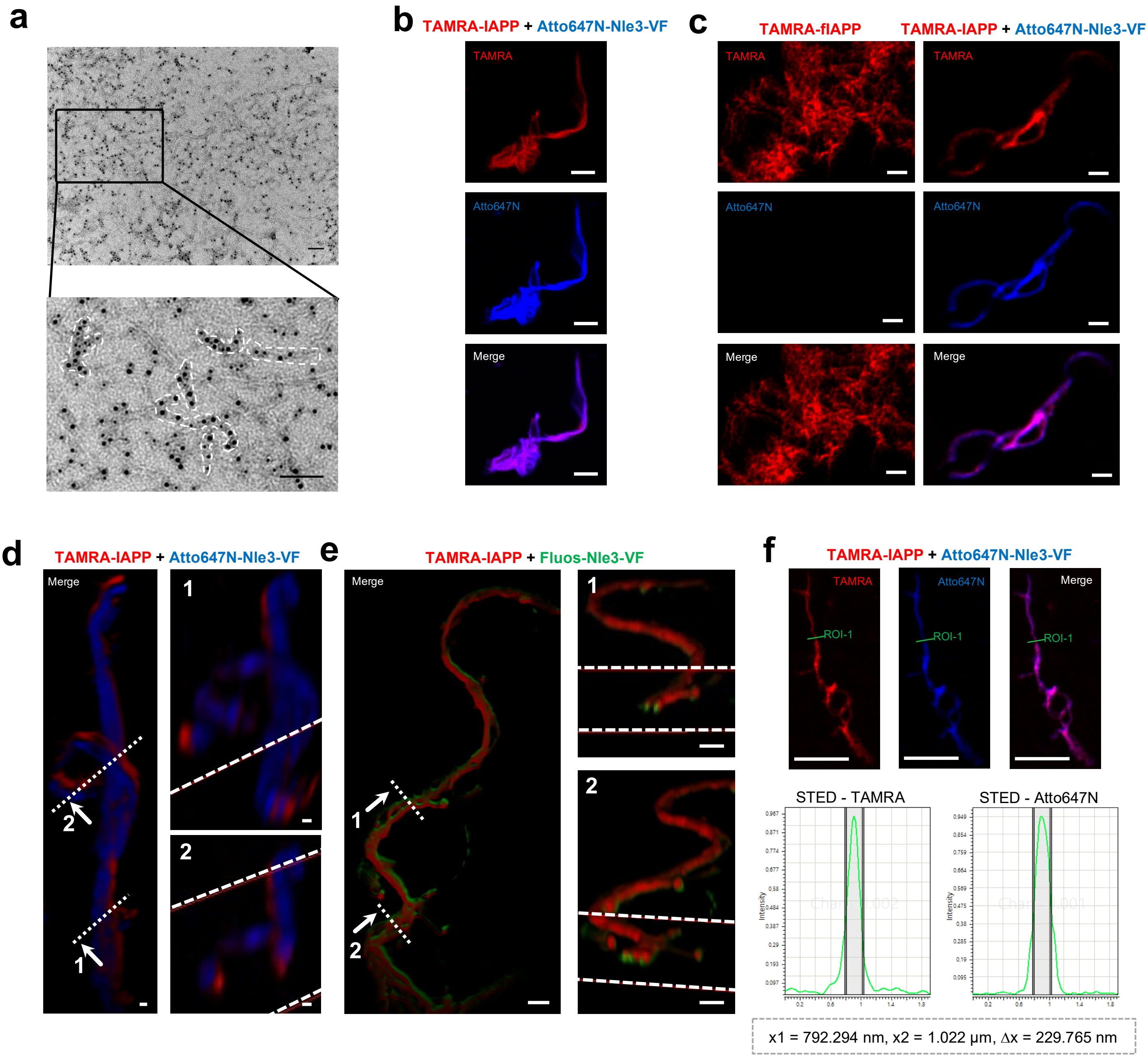


**Supplementary Fig. 11.** Additional TEM, CLSM, and STED evidence for IAPP/ACM co-assembly into nanofibers and supramolecular nanofiber bundles. **a** Immunogold TEM image of fibrils in aged IAPP/Nle3-VF mixture (IAPP, 16.5 µM; 1/2, 7 days aging) reveals fibrils which bind both the anti-fIAPP (IAPP fibril specific; 5 nm gold) and the anti-Aβ antibody (Nle3-VF; 10 nm gold). Highlighted areas depict fibrils which bind to both antibodies. Scale bars, 100 nm. Representative images from 4 biologically independent samples. **b** Confocal laser-scanning microscopy (CLSM) images of nanofiber co-assemblies in aged IAPP/Nle3-VF (1/2) mixture containing TAMRA-IAPP and Atto647N-Nle3-VF (10%) (IAPP(total) 16.5 µM, 7 day-aging). Scale bars, 5 µm. Image is representative of 11 assemblies found in one sample; consistent with 2PM of 3 biologically independent samples. **c** STED images of aged IAPP (fIAPP) or IAPP/Nle3-VF (1/2) mixture as indicated containing TAMRA-IAPP and Atto647N-Nle3-VF (10%) (IAPP(total) 16.5 µM, 7 days aging). Representative images from 4 similar biologically independent samples (fIAPP) or 11 nanofiber co-assemblies detected in one sample (IAPP/Nle3-VF); STED findings related to IAPP/Nle3-VF nanofiber co-assemblies were confirmed by 2PM in 3 biologically independent samples (see also Fig. 3d). Scale bars, 1 µm. **d** 3D reconstructions of z-stacks/still images of Supplementary Movie 1 of TAMRA-IAPP/Atto647N-Nle3-VF nanofiber co-assemblies shown in Fig. 3d. Arrows and dashed lines in the left panel indicate view of the sections

shown in the right panel. Scale bars, 1 µm. Representative images from reconstructions of z-stacks/still images of 8 nanofiber co-assemblies from 3 biologically independent samples. **e** 3D reconstructions of z-stacks/still images of Supplementary Movie 2 of TAMRA-IAPP/Fluos-Nle3-VF nanofiber co-assemblies found in the 2PM studies of Fig. 3d. Arrows and dashed lines in the left panel indicate view of the sections shown in the right panel. Scale bars, 5 µm. Representative images from reconstructions of z-stacks/still images of 8 nanofiber co-assemblies from 3 biologically independent samples. **f** Example of the estimation of the width of a TAMRA-IAPP/Atto647N-Nle3-VF nanofiber bundle found in the sample used for (**c**) determined with the "full-width-at half-maximum" values of an intensity-based line-profile plot. Top panel, STED image; green line indicates region of interest (ROI) chosen for measurement; scale bar, 5 µm. Bottom panel, intensity plots of Atto647N and TAMRA channels; $\Delta x$, heteromeric nanofiber bundle width measured at half-maximum of the peak height.

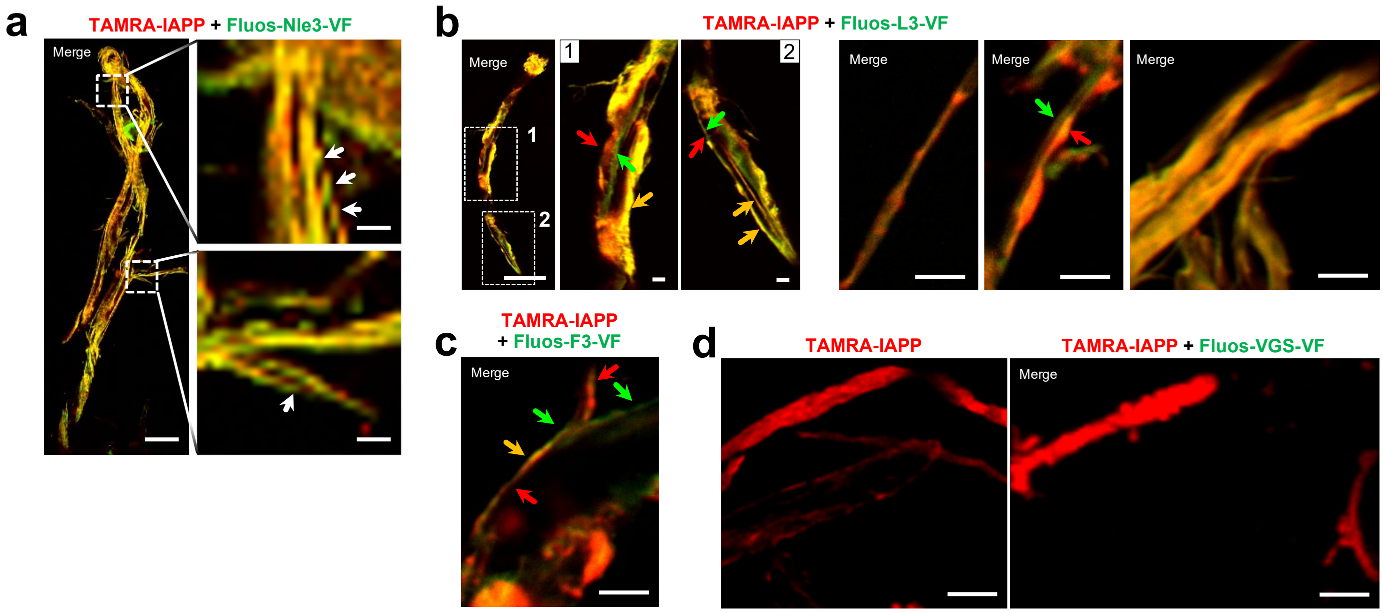


**Supplementary Fig. 12.** Additional 2PM evidence for supramolecular IAPP/ACM nanofiber co-assemblies. **a** 2PM image of a huge loop-like co-assembly (shown in Fig. 3g) found in aged TAMRA-IAPP/Fluos-Nle3-VF (1/2) mixtures (TAMRA-IAPP 16.5 μM; aging 6 days). In the insets, magnified image parts show μm-sized bicolored rods (widths ~1.7 μm, lengths ~5 μm) (short white arrows) as potential "building blocks" of braided parts of the co-assembly. Scale bars: 50 μm (insets 5 μm). Similar findings in two biologically independent samples (see Fig. 3g and 3h). **b** 2PM images of fibrous superstructures including tape-like heteromeric nanofiber bundles (right panel) found in aged TAMRA-IAPP/Fluos-L3-VF mixtures (TAMRA-IAPP 16.5 μM, 1/2, 7 day-aging). Colored arrows in the insets indicate fibrillar stacks of the two peptides arranged in parallel (red arrows, TAMRA-IAPP; green arrows, Fluos-L3-VF); or overlaying (yellow arrows). Scale bars: 50 μm in the image of the left panel and 5 μm for the magnified areas 1 and 2 and all images of the right panel. Representative images from one sample; consistent with findings in IAPP mixtures with other ACMs (see **a**,**c** and Fig. 3e-j). **c** 2PM images of heteromeric fibrous nanofiber bundles found in aged TAMRA-IAPP/Fluos-F3-VF mixtures (TAMRA-IAPP 16.5 μM, 1/2, 7 day-aging); color code for arrows as in **a**; scale bars, 5 μm; representative image from one sample. **d** 2PM images of fibrillar assemblies found in aged TAMRA-IAPP (TAMRA-fIAPP) (16.5 μM, 7 day-aged) (left) and its aged mixture with the non-inhibitor Fluos-VGS-VF (1/2, 7 day-aged) (right). These latter mixtures consisted mostly of TAMRA-fIAPP bundles. Scale bars, 5 μm. Representative images from 3 (TAMRA-fIAPP) and 2 (TAMRA-IAPP/Fluos-VGS-VF) biologically independent samples.

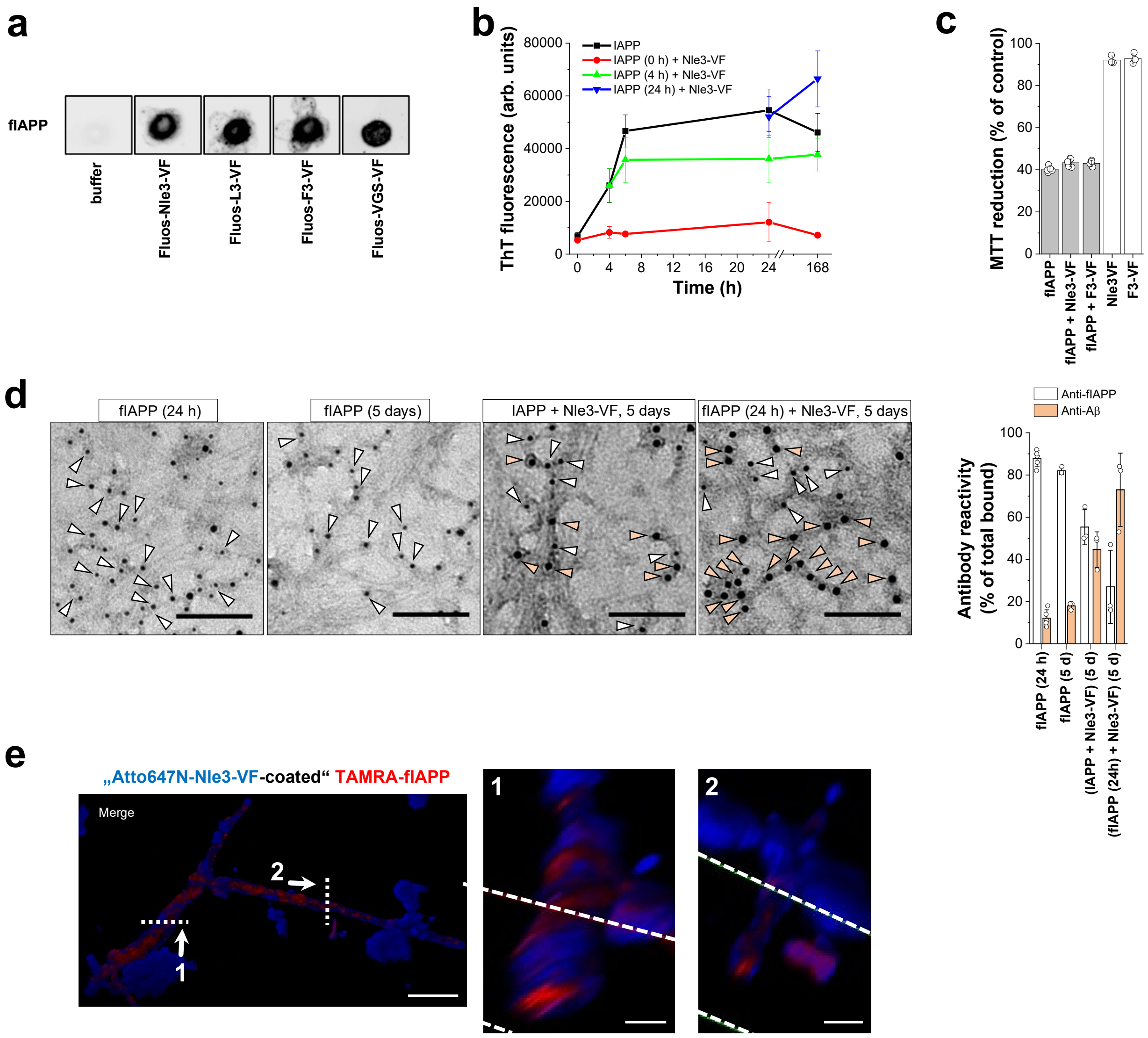


**Supplementary Fig. 13.** ACM-coated fIAPP are distinct from ThT-invisible and non-toxic hf-IAPP/ACM and their formation is not a major reason for ACM inhibitory activity on IAPP amyloid self-assembly. **a** Dot blot analysis reveals that both ACMs and the non-inhibitor VGS-VF bind fIAPP. Membranes containing spotted fIAPP (40 μg) were probed with N-terminal fluorescein-labeled peptides (Fluos-ACMs and Fluos-VGS-VF, 0.2 μM). Representative results from 2 (Nle3-VF, L3-VF, and F3-VF) or 3 (VGS-VF) independent assays are shown. **b** Addition of Nle3-VF to already nucleated IAPP fibrillogenesis does not affect the amount of ThT-reactive fibrils. ThT binding was measured in IAPP (16.5 μM) alone or its mixtures with Nle3-VF (33 μM) at the indicated time points before and after Nle3-VF addition. Data are means ± SD from 3 independent assays. **c** Addition of ACMs to preformed fIAPP does not affect fIAPP cytotoxicity. ACMs (Nle3-VF and F3-VF; 33 μM) were added to preformed fIAPP (16.5 μM, 7 days aged). Following co-incubation for 1 day, fIAPP alone, its mixtures with the ACMs, and ACMs alone were added to RIN5fm cells (fIAPP, 500 nM). Cell damage was determined via MTT reduction. Data of fIAPP and its mixtures are means ± SD of 6 wells from 2 independent assays (n=3 technical replicates each). Data of ACMs alone are from 1 assay (n=3 technical replicates); additional data on the lack of cytotoxic effects of the ACMs are in Supplementary Fig. 5c. **d** Immunogold TEM indicates a clear difference between the antibody binding ability of fibrils in aged IAPP/Nle3-VF mixtures (hf-IAPP/Nle3-VF) and the fibrils in “Nle3-VF-coated” fIAPP solutions. Shown are immunogold TEM images of fIAPP solutions (24 h or 5 day-aged), IAPP/Nle3-VF mixtures (1/2; IAPP 16.5 μM, 5 day-aged), and Nle3-VF-coated fIAPP as indicated. Nle3-VF-coated fIAPP was made by adding Nle3-VF (33 μM) to preformed fIAPP (IAPP (16.5 μM) aged for 24 h) and co-incubating for 5 days. Representative assemblies from 5 or 3 fields of view of one grid of each solution are shown; images of fIAPP and IAPP/Nle3-VF solutions are representative

from 4 biologically independent samples (see also Fig. 3a). fIAPP was detected by anti-fIAPP specific antibody (5 nm gold nanoparticles; white arrowheads) and Nle3-VF by anti-Aβ antibody (10 nm gold nanoparticles; orange arrowheads) exhibiting a 10-20% NSB to fIAPP (see antibody binding quantification graph; right side). Scale bars: 100 nm. On the right side, the quantification of antibody binding of each of the solutions expressed as antibody reactivity is presented ("d", days). Antibody reactivities were calculated as % of total bound nanoparticles in several fields of view of one grid for each sample. They are means $\pm$ SD from 50, 53, 21, and 115 fibrillar assemblies present in the grid of fIAPP (24 h), fIAPP (5 days), "(IAPP + Nle3-VF) (5 days)", and "(IAPP (24 h) + Nle3-VF (5 days))", respectively. The results indicate that Nle3-VF-coated fIAPP bound more anti-Aβ antibody than hf-IAPP/Nle3-VF. **e** 2PM examination of Nle3-VF-coated fIAPP reveal a distinct morphology that is different from hf-IAPP/Nle3-VF. In contrast to hf-IAPP/Nle3-VF, Nle3-VF-coated fIAPP consisted of fIAPP bundles randomly covered by large amorphous Fluos-Nle3-VF aggregates. 3D reconstructions of its z-stacks/still images of Supplementary Movie 3 are shown. Representative image from 3 similar biologically independent samples. Arrows and dashed lines in the left panel indicate view of the sections shown in right panel. Scale bars, 20 μm for the image in the left panel and 5 μm for image sections 1 and 2.

**a**

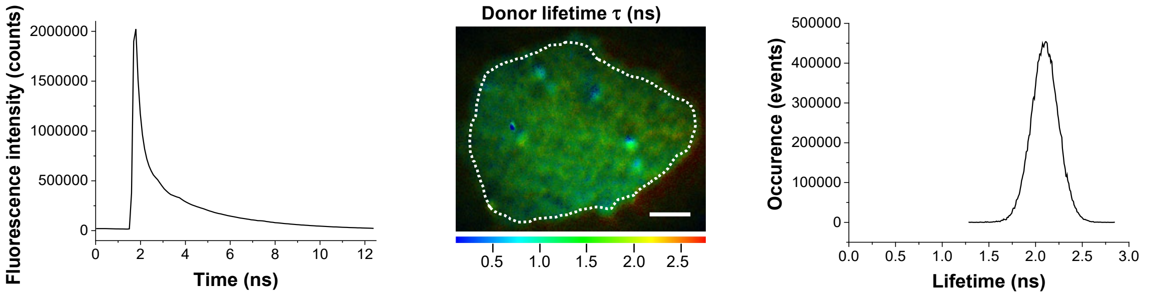


**b**

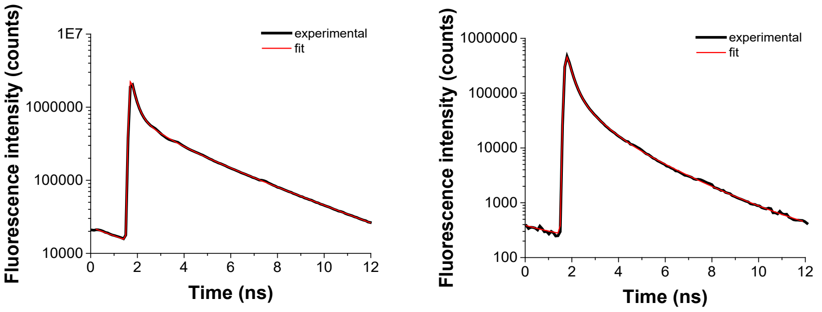


**Supplementary Fig. 14. Fluorescence decay, FLIM image, and donor lifetime distribution of Fluos-Nle3-VF (donor) alone used in the FLIM-FRET analysis of TAMRA-IAPP/Fluos-Nle3-VF nanofiber co-assembly of Fig. 3j and experimental and fitted decay curves of Fluos-Nle3-VF in absence or presence of TAMRA-IAPP. a** Left panel, fluorescence decay curve of Fluos-Nle3-VF alone incubated under the same experimental conditions (Fluos-Nle3-VF 33 μM, 6 day-aged) as the TAMRA-IAPP/Fluos-Nle3-VF (1/2) mixture containing the co-assembly of Fig. 3g which was analyzed in Fig. 3j. Middle panel, FLIM image showing the Fluos-Nle3-VF lifetime in absence of acceptor (TAMRA-IAPP); lifetime range indicated by the colored bar. Scale bar: 10 μm. Right panel, Fluos-Nle3-VF lifetime distribution in absence of TAMRA-IAPP. Similar data were obtained with 2 biologically independent Fluos-Nle3-VF samples examined following aging for 0 and 48 h (1$^{st}$ sample) or for 6 days (2$^{nd}$ sample) (see Supplementary Fig. 16 and 17). **b** Experimental and fitted fluorescence decay curves of Fluos-Nle3-VF in the absence (left panel) or presence (right panel) of TAMRA-IAPP in the TAMRA-IAPP/Fluos-Nle3-VF nanofiber co-assembly of Fig. 3j (TAMRA-IAPP 16.5 μM, Fluos-Nle3-VF 33 μM, 6 day-aged).

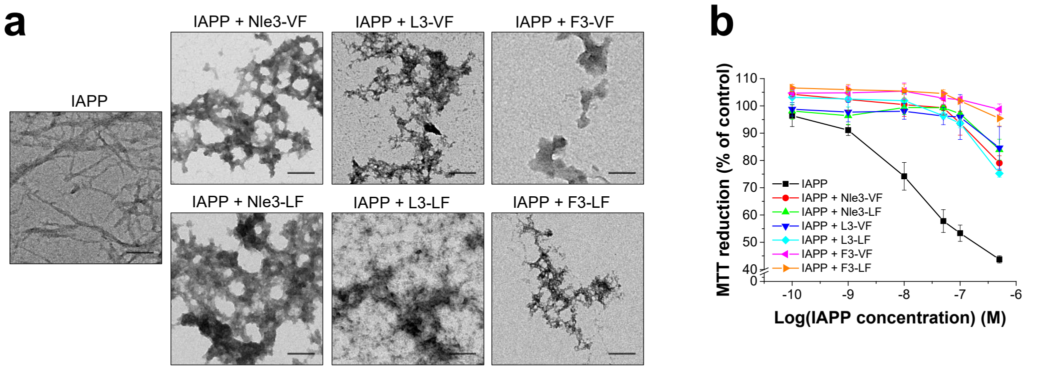


**Supplementary Fig. 15.** Early IAPP/ACM co-assemblies are amorphous and non-cytotoxic. **a** Early IAPP/ACM co-assemblies consist mostly of amorphous aggregates. TEM analysis of 24 h aged incubations of IAPP (16.5 μM) and its mixtures with ACMs (33 μM). Scale bars: 100 nm. Data are representative from 3 or 2 biologically independent samples of IAPP or IAPP/Nle3-VF, respectively, 2 similar biologically independent samples of IAPP/Nle3-LF or IAPP-L3-VF, and one sample of each of the remaining IAPP/ACM mixtures. **b** Early non-fibrillar IAPP/ACM co-assemblies are non-cytotoxic. Incubations of IAPP (16.5 μM) and its mixtures with ACMs (1/2; 24 h-aged) were added to RIN5fm cells and cell damage was determined via MTT reduction (means ± SD, 3 independent assays, n=3 technical replicates each).

Supplementary Fig. 16

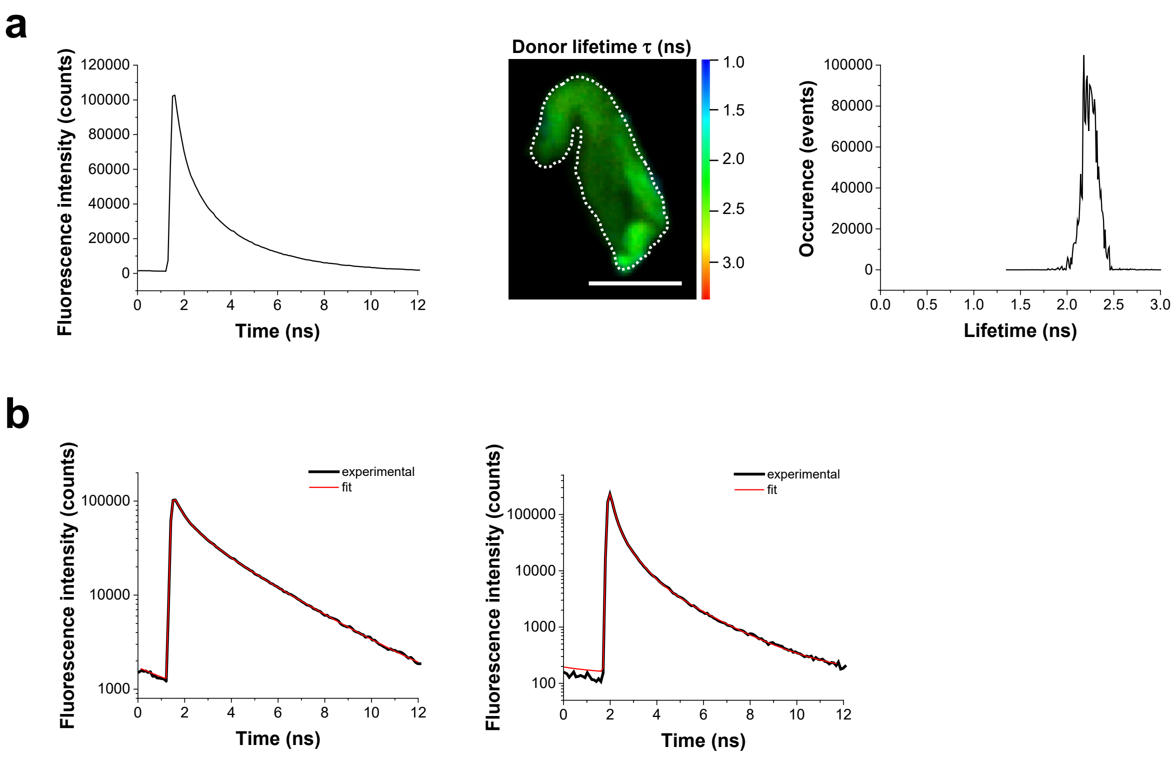


**Supplementary Fig. 16. Fluorescence decay, FLIM image, and donor lifetime distribution of Fluos-Nle3-VF (donor) alone used in the FLIM-FRET analysis of TAMRA-IAPP/Fluos-Nle3-VF nanofiber co-assembly of Fig. 4d and experimental and fitted decay curves of Fluos-Nle3-VF in absence or presence of TAMRA-IAPP (acceptor). a** Left panel, fluorescence decay curve of Fluos-Nle3-VF alone incubated under the same experimental conditions (Fluos-Nle3-VF 33 μM, 48 h-aged) as the TAMRA-IAPP/Fluos-Nle3-VF (1.65 μM/33 μM, 48 h-aged) mixture containing the co-assembly of Fig. 4c which was analyzed in Fig. 4d. Middle panel, FLIM image showing the Fluos-Nle3-VF lifetime in absence of TAMRA-IAPP; lifetime range indicated by the colored bar. Scale bar: 10 μm. Right panel, Fluos-Nle3-VF lifetime distribution in absence of TAMRA-IAPP. Similar results were obtained from 2 biologically independent Fluos-Nle3-VF samples examined following aging for 0 and 48 h (1$^{st}$ sample) or for 6 days (2$^{nd}$ sample) (see Supplementary Fig. 14 and 17). **b** Experimental and fitted fluorescence decay curves of Fluos-Nle3-VF in the absence (left panel) or presence (right panel) of TAMRA-IAPP in the TAMRA-IAPP/Fluos-Nle3-VF nanofiber co-assembly of Fig. 4d.

Supplementary Fig. 17

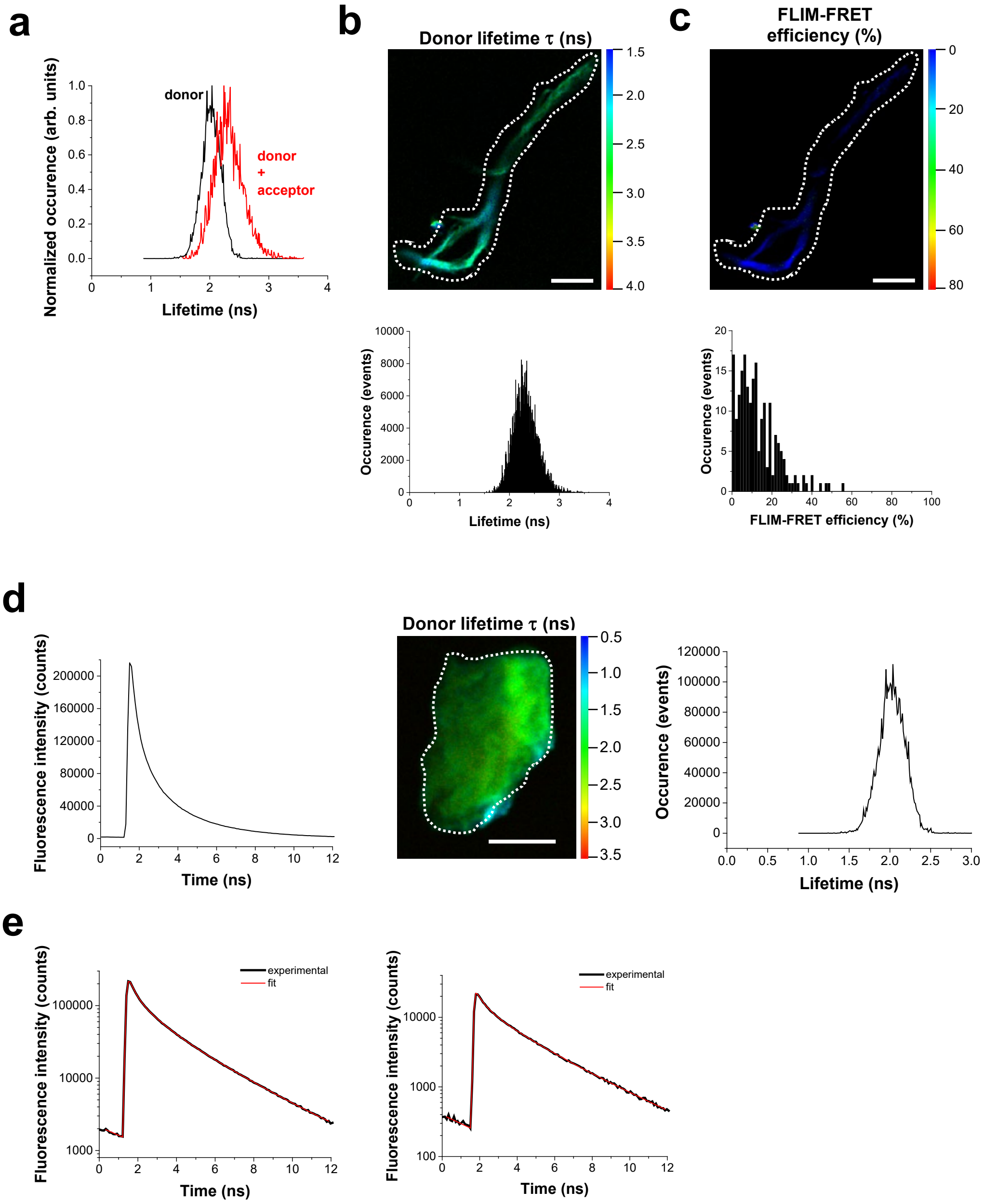


**Supplementary Fig. 17.** FLIM-FRET analysis of fibrillar TAMRA-fIAPP/Fluos-Nle3-VF co-assemblies found in Fluos-Nle3-VF (33 μM) following addition of preformed TAMRA-fIAPP (10%) revealed no appreciable FLIM-FRET events. **a** Fluorescence lifetime of donor (Fluos-Nle3-VF) without or with acceptor (TAMRA-fIAPP). **b** Upper part, FLIM image of the fibrillar co-assembly showing donor lifetime; lifetime as indicated; scale bar, 10 µm. Lower part, donor fluorescence lifetime distributions (~2.3 ns) in the fibrillar co-assembly. **c** Upper part, FLIM-FRET efficiency (%) observed in the fibrillar co-assembly; efficiency distribution as indicated; scale bar, 10 µm. Lower part, FLIM-FRET efficiency distribution in the fibrillar co-assembly (<30%). Data in **a**-**c** are from 1 experiment. **d** Left panel, fluorescence decay curve of Fluos-Nle3-VF alone. Of note, Fluos-Nle3-VF was incubated under the same experimental conditions (Fluos-Nle3-VF 33 µM, 0 h) as the fTAMRA-IAPP/Fluos-Nle3-VF (3.3 μM/33 μM, 0 h) mixture containing the co-assembly analyzed in **a**-**c**. Middle panel, FLIM image showing Fluos-Nle3-VF lifetime in absence of TAMRA-fIAPP; lifetime range indicated by the coloured bar. Scale bar: 10 µm. Right panel, Fluos-Nle3-VF lifetime distribution in absence of TAMRA-fIAPP (see also **a**). Similar results were obtained with 2 biologically independent Fluos-Nle3-VF samples examined following aging for 0 and 48 h ($1^{st}$ sample) and for 6 days ($2^{nd}$ sample) (see Supplementary Fig. 14 and 16). **e** Experimental and fitted fluorescence decay curves of Fluos-Nle3-VF in the absence (left panel) or presence (right panel) of TAMRA-fIAPP in the TAMRA-fIAPP/Fluos-Nle3-VF fibrillar co-assembly of **b.**

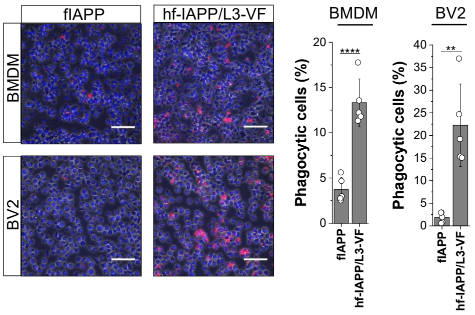


**Supplementary Fig. 18.** IAPP/L3-VF-nanofibers are much more efficiently phagocytosed than fIAPP by primary murine BMDMs and cultured murine BV2 microglia. Left panel, representative microscopic images of BMDMs or BV2 cells as indicated following incubation (6 h, 37°C) with TAMRA-fIAPP (3.3 μM) (left side) or hf-TAMRA-IAPP/L3-VF (3.3 μM) (right side) as indicated (compare with Fig. 4h); red dots indicate TAMRA-IAPP; scale bars, 100 μm. Right panel, amounts of BMDM (mid) or BV2 (right) cells (% of total) that phagocytosed fIAPP and hf-IAPP/L3-VF. Data are means ± SD from 5 biologically independent fIAPP samples and 5 biologically independent hf-IAPP/L3-VF samples studied in one cell assay with each well analyzed in 3 fields of view; ****P<0.0001 (P=8.4E-05) and **P<0.01 (P=0.0011) for fIAPP versus hf-IAPP/L3-VF in BMDM and BV2 cells, respectively as indicated (unpaired t-test (2-sided)).

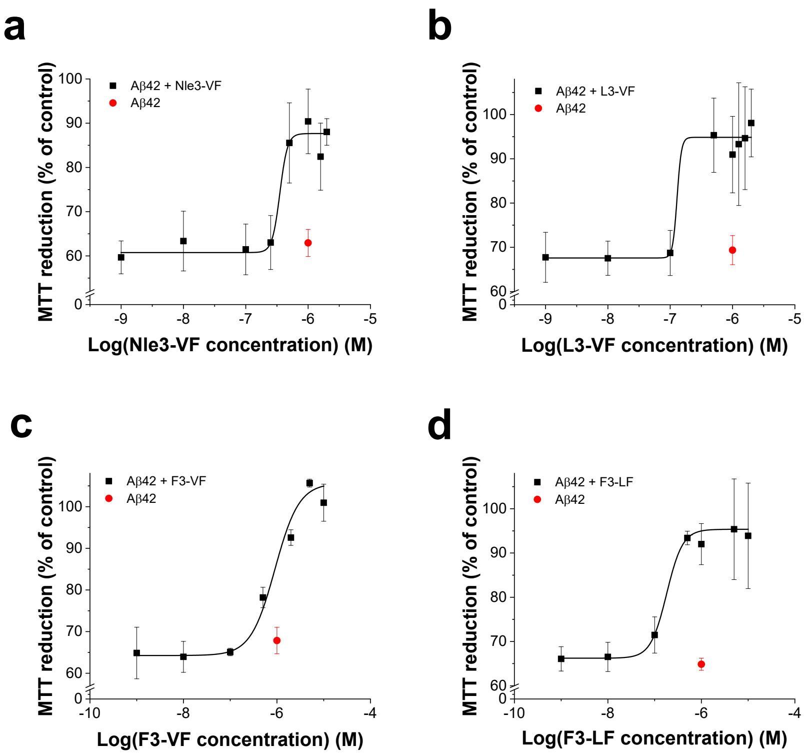


**Supplementary Fig. 19.** Determination of $IC_{50}$ values of inhibitory effects of ACMs on formation of cell-damaging Aβ42 assemblies. PC12 cells were incubated with 6-day aged Aβ42 alone (1 μM) or its mixtures with different molar ratios of ACMs and cell damage was determined by the MTT reduction assay for mixtures of Aβ42 with Nle3-VF (**a**), L3-VF (**b**), F3-VF (**c**) and F3-LF (**d**); red symbols show effects of Aβ42 alone. Data and $IC_{50}$ values are means ± SD from 3 independent assays (n=3 technical replicates each).

Supplementary Fig. 20

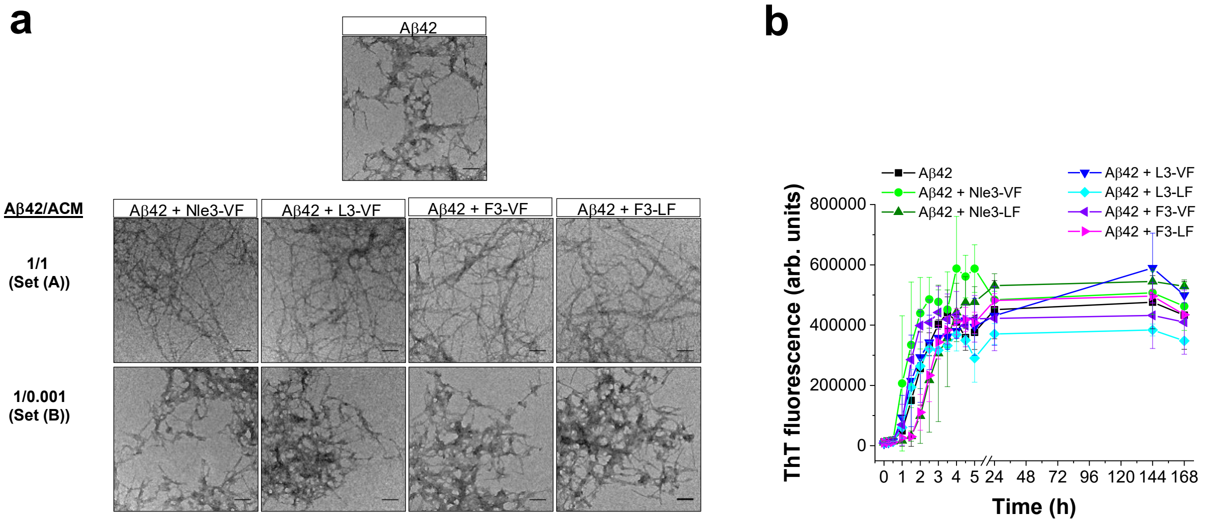


**Supplementary Fig. 20.** Formation of long fibrils in Aβ42/ACM mixtures is linked to ACM inhibitory effect on Aβ42 fibrillogenesis. **a** TEM images of two 6 day-aged sets of mixtures, i.e. the Aβ42/ACM 1/1 mixtures (set (A); full inhibition of Aβ42 fibrillogenesis according to ThT binding (Fig. **6a**)) and the Aβ42/ACM 1/0.001 mixtures (set (B); no inhibition of fAβ42 fibrillogenesis according to ThT binding (shown in **b**) as compared to 6 day-aged Aβ42 alone (Aβ42, 5 μM). All four “ThT-negative” mixtures of set (A) consisted of 2-4-fold longer fibrils than Aβ42 aged under the same conditions, which consisted of “ThT-positive” fAβ42 (Fig. 6d). By contrast, all four ThT-positive mixtures of set (B) consisted of fibrils of identical appearance and widths to fAβ42. Scale bars: 100 nm. Images of set (A) are representative of 3 (Aβ42/Nle3-VF) or 2 (Aβ42/L3-VF, Aβ42/F3-VF and Aβ42/F3-LF) biologically independent samples. Images of set (B) represent findings from one sample for each peptide mixture analyzed in different fields of view. **b** Aβ42 amyloid self-assembly is not affected when an Aβ42/ACM molar ratio of 1/0.001 is used. Aβ42 fibrillogenesis (5 μM) alone or with each of the ACMs (5 nM) assessed by ThT binding (means ± SD, 3 independent assays). Of note, TEM images in **a** are from 6 day-aged solutions made as in **b** but without ThT.

Supplementary Fig. 21

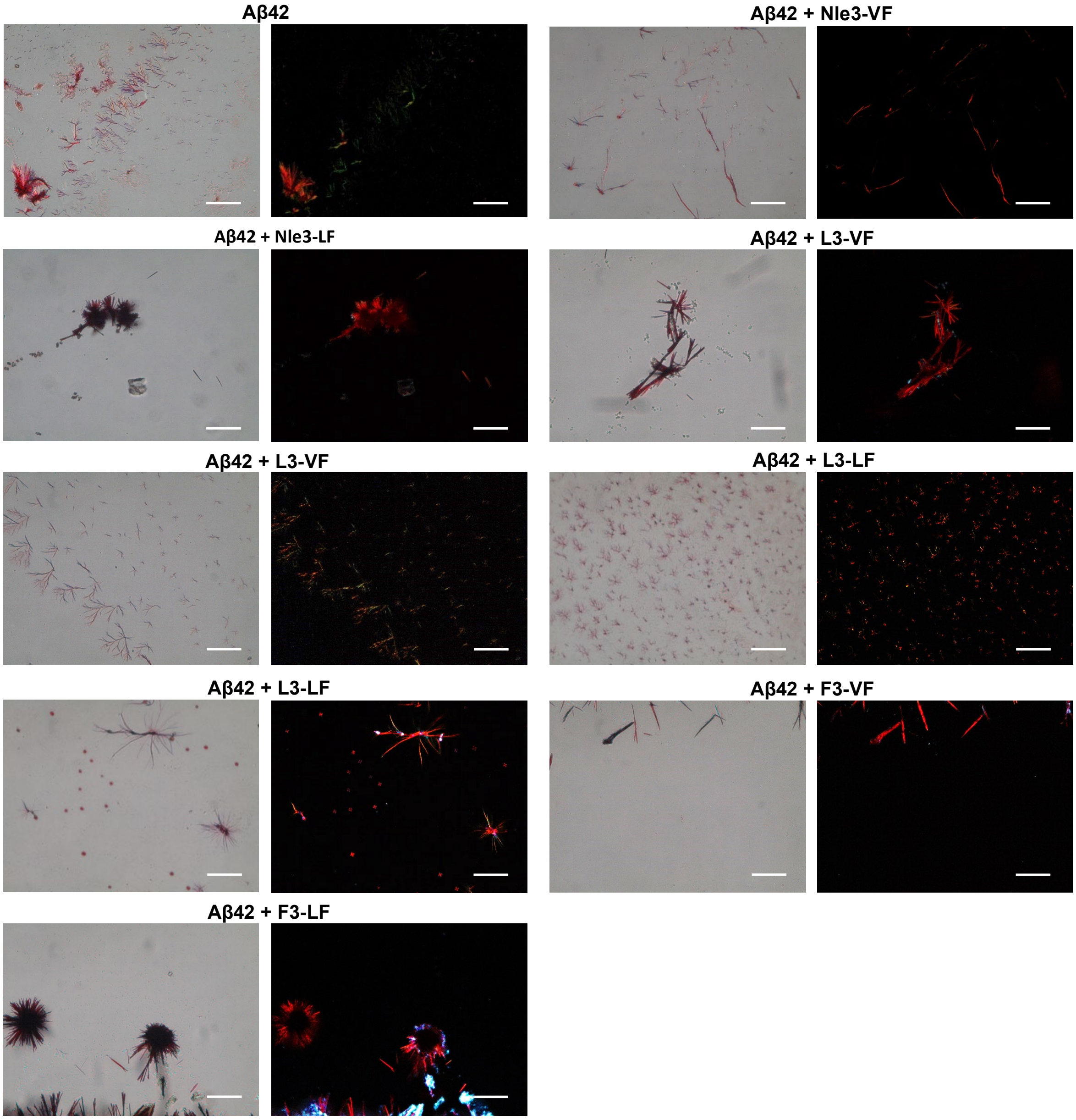


**Supplementary Fig. 21. Most of the ThT-invisible fibrillar assemblies in Aβ42/ACM mixtures do not exhibit birefringence under polarized light when stained with CR.** Microscopic images from bright field or between crossed polarizer/analyzer examination of fAβ42 (5 μM Aβ42, 6 day-aged) and its mixtures with various ACMs (1/1, 6 day-aged) consisting mostly of fibrillar assemblies (Fig. 6d) are shown. Images are representative from 3 biologically independent fAβ42 samples or from one sample for each of the Aβ42/ACM mixtures examined in various different fields of view. Solutions were made as for ThT binding assay (without ThT) (Fig. 6a). In contrast to fAβ42, most of the fibrillar assemblies in Aβ42/ACM solutions were not birefringent when examined under polarized light. Only in the case of Aβ42/L3-VF and Aβ42/L3-LF solutions, we also observed some green/yellow birefringent fibrillar assemblies. Scale bars, 10 μm.

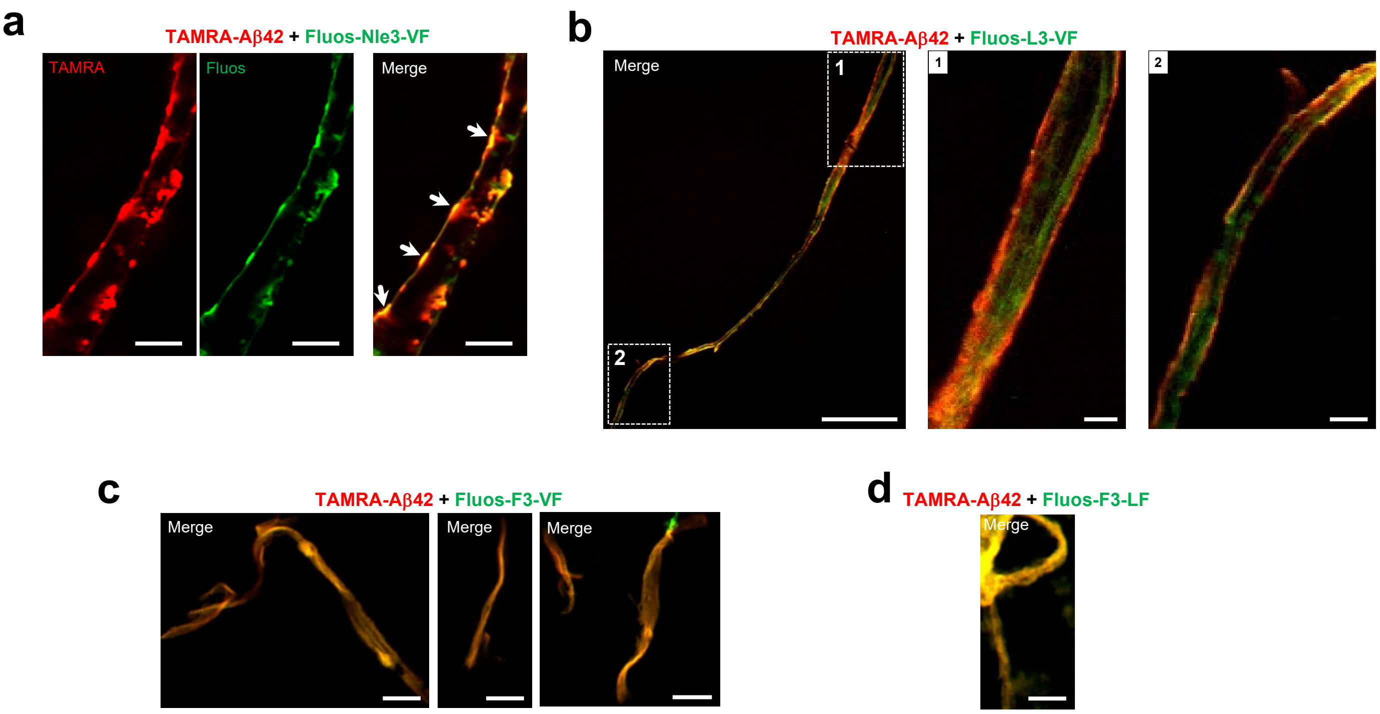


**Supplementary Fig. 22.** 2PM images of diverse fibrous Aβ42/ACM superstructures. **a** Supramolecular Aβ42/Nle3-VF nanofiber co-assembly containing 50%TAMRA-Aβ42/Fluos-Nle3-VF (merged image presented in Fig. 6e) is shown here with each channel (CH) (CH1 (TAMRA) and CH2 (Fluos)) separately and merged; see also Supplementary Movie 5. Scale bars, 10 µm. Consistent findings in 3 biologically independent samples. **b** Huge Aβ42/L3-VF ribbon-/nanotube-like nanofiber co-assembly (TAMRA-Aβ42/Fluos-L3-VF (50%); Aβ42(total), 5 µM; 1/2, 6 days). Scale bars, 100 µm and 10 µm in magnified areas 1 and 2. Consistent findings in 2 biologically independent samples. **c, d** Aβ42/F3-VF and Aβ42/F3-LF ribbon-like nanofiber co-assemblies (TAMRA-Aβ42/Fluos-ACM (50%); Aβ42(total) 5 µM, 1/2, 6 days) as indicated. Scale bars, 10 µm. Consistent findings in 2 biologically independent Aβ42/F3-VF samples; Aβ42/F3-LF images are from one sample.

Supplementary Fig. 23

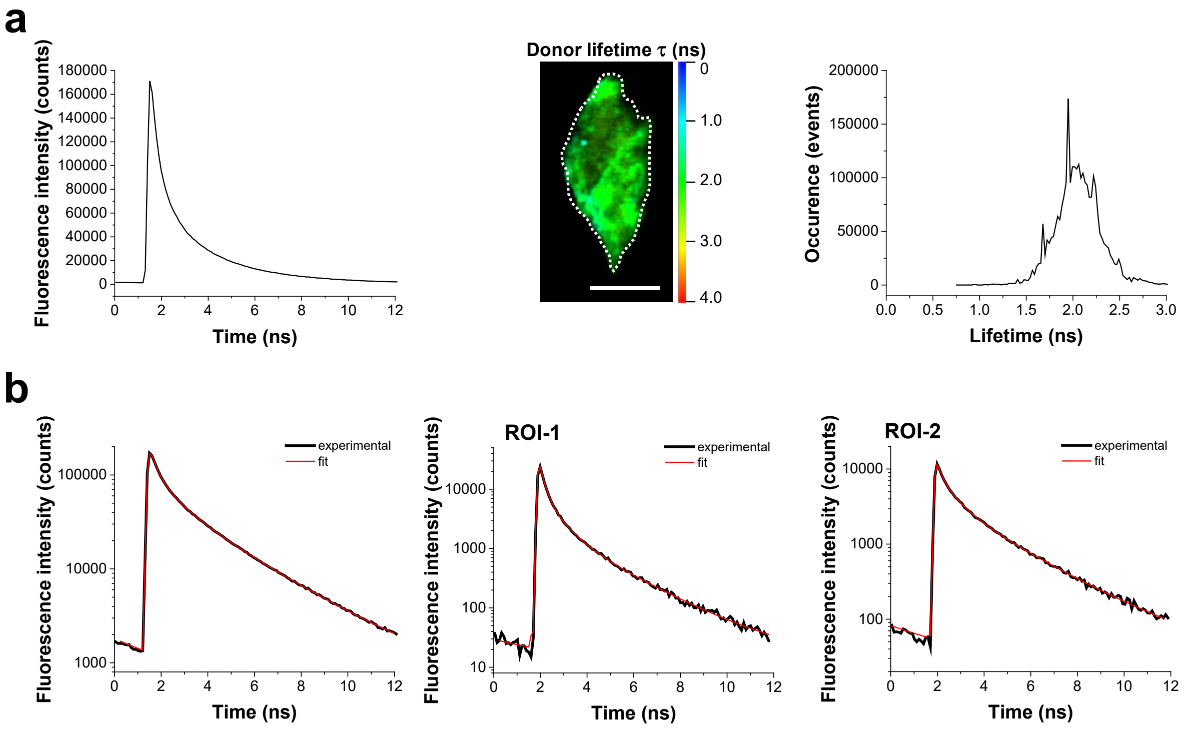


**Supplementary Fig. 23. Fluorescence decay, FLIM image, and donor lifetime distribution of Fluos-Nle3-VF (donor) alone used in the FLIM-FRET analysis of TAMRA-Aβ42/Fluos-Nle3-VF nanofiber co-assembly of Fig. 6f and experimental and fitted decay curves of Fluos-Nle3-VF in absence or presence of TAMRA- Aβ42. a** Left panel, fluorescence decay curve of Fluos-Nle3-VF incubated under the same experimental conditions as the TAMRA-Aβ42/Fluos-Nle3-VF mixture containing the co-assembly of Fig. 6e which was analyzed in Fig. 6f. Middle panel, FLIM image showing the Fluos-Nle3-VF lifetime in absence of acceptor (TAMRA-Aβ42); lifetime range indicated by the colored bar. Scale bar: 10 μm. Right panel, Fluos-Nle3-VF lifetime distribution in absence of TAMRA-Aβ42. Consistent results were obtained with 3 similar biologically independent Fluos-Nle3-VF samples (see Supplementary Fig. 14 and 16). **b** Experimental and fitted fluorescence decay curves of Fluos-Nle3-VF in the absence (left panel) or presence of TAMRA-Aβ42 in ROI-1 (middle panel) and ROI-2 (right panel) of hf-TAMRA-Aβ42/Fluos-Nle3-VF of Fig. 6f.

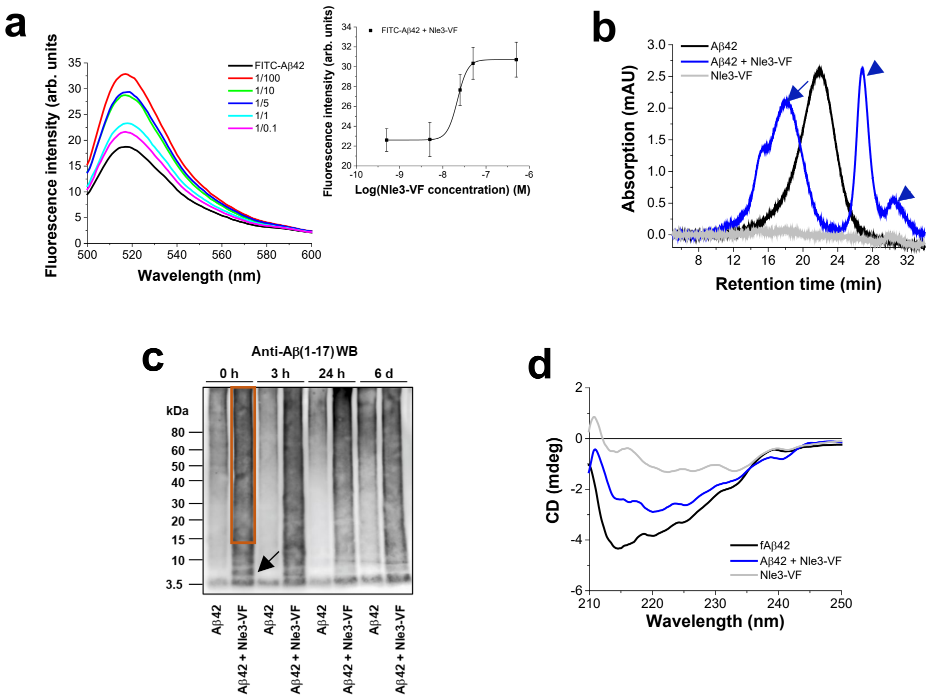


**Supplementary Fig. 24.** Characterization of Aβ42/ACM interactions and hetero-complexes by fluorescence spectroscopy, size-exclusion chromatography (SEC), cross-linking, and far-UV CD spectroscopy. **a** Nle3-VF binds Aβ42 with low nanomolar affinity as determined by fluorescence spectroscopic titrations. Fluorescence spectra of FITC-Aβ42 (5 nM) and its mixtures with Nle3-VF (pH 7.4) at indicated molar ratios (data from one representative binding assay from n=3 independent assays). Inset, binding curve; data means ± SD from 3 independent assays; app. $K_D$, 14.5 ± 8.0 (Table 2). **b** Characterization of hf-Aβ42/Nle3-VF via SEC. Chromatograms of aged (6 days) Aβ42 (5 µM), Nle3-VF (5 µM), and their 1/1 mixture (5 µM each) consisting mostly of ThT-invisible fibrils (Fig. 6d) are shown (mAU, milli-absorbance units). Arrow indicates high MW hetero-assemblies; arrowheads indicate Aβ42 and Nle3-VF monomers. Representative results from 2 independent experiments. **c** Kinetics of Aβ42/Nle3-VF co-assembly as followed by cross-linking with glutaraldehyde. This was performed in solutions of Aβ42, Aβ42/Nle3-VF (1/2), and Nle3-VF at different incubation time points in combination with NuPAGE and WB with anti-Aβ(1-17) antibody (6E10) which recognizes Aβ42 but not the ACM. Orange box marks smear of unresolved bands corresponding of medium-to-high MW hetero-assemblies; arrow indicates hetero-dimers. Results are representative of 2 independent experiments. **d** hf-Aβ42/Nle3-VF have less β-sheet structure than fAβ42. Far-UV CD spectra of aged (6 days) Aβ42 (5 µM), Nle3-VF (5 µM) and their 1/1 mixture (5 µM each) are shown. Under these experimental conditions Aβ42 and Aβ42/Nle3-VF mixtures consisted mostly of fibrils (Fig. 6d). Results are from one experiment; comparable results were obtained with Aβ42/L3-VF (1/1).

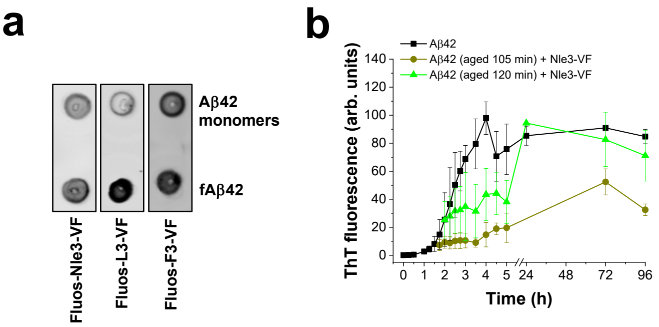


**Supplementary Fig. 25.** Binding of ACMs to preformed fAβ42 does not convert fAβ42 into ThT-invisible heteromeric nanofibers. **a** Dot blot analysis shows that ACMs bind to fAβ42. Membranes containing spotted fAβ42 or Aβ42 monomers (10 μg each) were probed with various Fluos-ACMs (2 μM); results are representative from 2 independent assays. **b** Addition of Nle3-VF to already nucleated fAβ42 fibrillogenesis does not affect amounts of already formed ThT-positive fibrils. ThT binding was measured in Aβ42 (5 μM) alone or its mixtures with Nle3-VF (5 μM) at the indicated time points before and after Nle3-VF addition. Data means ± SD (3 independent assays except for the 24 h time point of “Aβ42 (aged 120 min) + Nle3-VF” (1 assay)).

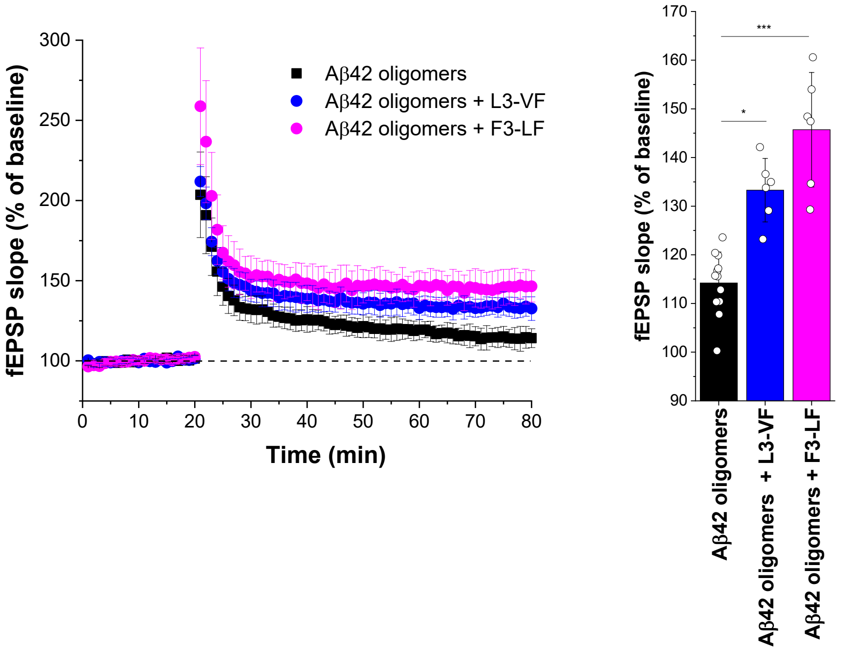


**Supplementary Fig. 26. L3-VF and F3-LF suppress LTP impairment mediated by preformed Aβ42 oligomers in murine hippocampal slices ex vivo.** Left, time course of synaptic transmission; means ± SD (n=12 for Aβ42 oligomers and n=6 for Aβ42 oligomers/ACM mixtures (1/10); (Aβ42 oligomers (50 nM), 24 h aged, 30°C). Right, LTP values: averages from the last 10 min of recording; data, means ± SD (n, see above); ***P<0.001 (P=0.0003) and *P<0.05 (P=0.0152) versus Aβ42 oligomers as indicated (Kruskal-Wallis test with Dunn's multiple comparisons test).

Supplementary Fig. 27

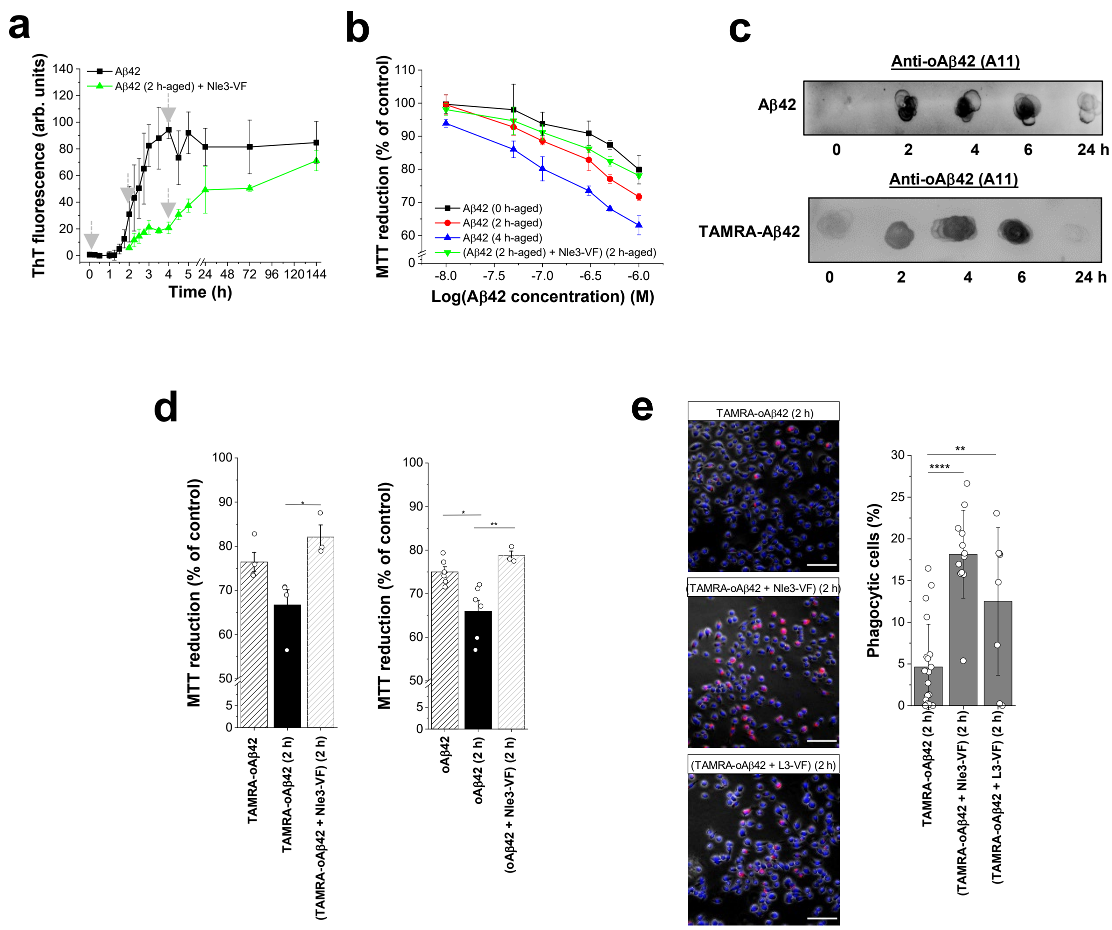


**Supplementary Fig. 27. Addition of ACMs to preformed toxic Aβ42 oligomers (“oAβ42”) results in a suppression of fibrillogenesis and cytotoxicity and an increase of Aβ42 phagocytosis by BV2 microglia. a** Addition of Nle3-VF to preformed toxic oAβ42 oligomers (2 h-aged Aβ42 (5 μM); presence and cytotoxicity of oligomers confirmed by Dot blot analysis with A11 antibody and the MTT reduction assay, see **b**,**c**) results in a delay of fibrillogenesis as determined by the ThT binding assay (means ± SD, 3 independent assays). ThT binding measured in Aβ42 (5 μM) alone or its mixtures (1/1) with Nle3-VF before and after Nle3-VF addition at the indicated time points. Grey arrows indicate time points of addition of aliquots of solutions running in parallel to the above solutions to PC12 cells for the MTT reduction assay shown in **b**. **b** Addition of Nle3-VF to preformed cytotoxic oAβ42 (2 h-aged Aβ42 (5 μM); see **a**) suppresses formation of more toxic species as determined by the MTT reduction assay (means ± SD, 3 independent assays, n=3 technical replicates each). Aliquots of solutions (made as in **a** but without ThT) were added (1 μM) to PC12 cells and cellular MTT reduction was measured at the indicated incubation time points (0, 2, and 4 h; grey arrows in **a**). **c** Dot blot analysis of Aβ42 (top) and TAMRA-Aβ42 (bottom) (5 μM; made as above but without ThT) at different time points of incubation using anti-oligomer A11 antibody indicates the presence of significant amounts of (TAMRA-)Aβ42 oligomers in 2-6 h aged solutions. Results are representative from 2 (Aβ42) or 3 (TAMRA-Aβ42) independent assays. As the 4 and 6 h-aged solutions contained large amounts of fibrils as well according to the ThT assay shown in **a**, the 2 h-aged (TAMRA-)Aβ42 solutions were used to study whether direct addition of ACMs to preformed oligomers (“(TAMRA-)oAβ42”) might affect their cytotoxicity and phagocytosis (see **d** and **e**). **d** oAβ42 and TAMRA-oAβ42 exhibit similar cell-damaging properties and Nle3-VF (1/1) suppresses their cytotoxicity. Left panel, addition of Nle3-VF to preformed TAMRA-oAβ42 suppresses cytotoxicity as determined by the MTT reduction assay (means ± SD from 4 (TAMRA-oAβ42 and “TAMRA-oAβ42 (2 h)”) or 3 (“(TAMRA-oAβ42 + Nle3-VF) (2 h)”) independent assays, n=3 technical

replicates each); *P<0.05 (P=0.02001) for "(TAMRA-oAβ42 + Nle3-VF) (2 h)" versus "TAMRA-oAβ42 (2 h)" as indicated (one-way ANOVA & Bonferroni). Aliquots of TAMRA-oAβ42 alone (2 h-aged TAMRA-Aβ42), TAMRA-oAβ42 aged for additional 2 h ("TAMRA-oAβ42 (2 h)"), and TAMRA-oAβ42 following addition of Nle3-VF (1/1) and incubation for 2 h ("(TAMRA-oAβ42 + Nle3-VF) (2 h)") were added (1 μM) to PC12 cells. Right panel, the corresponding oAβ42 data are shown for comparison. Addition of Nle3-VF to preformed oAβ42 suppresses cytotoxicity as determined by the MTT reduction assay. Aliquots of oAβ42 alone (2 h-aged Aβ42), oAβ42 aged for additional 2 h ("oAβ42 (2 h)"), and oAβ42 following addition of Nle3-VF (1/1) and incubation for 2 h ("(oAβ42 + Nle3-VF) (2 h)") were added (1 μM) to PC12 cells. Results are means ± SD from 6 (oAβ42 and "oAβ42 (2 h)") or 3 ("(oAβ42 + Nle3-VF) (2 h)") independent assays, n=3 technical replicates each); *P<0.05 (P=0.0129) for "oAβ42 (2 h)" versus oAβ42 and **P<0.01 (P=0.00477) for "(oAβ42 + Nle3-VF) (2 h)" versus "oAβ42 (2 h)" as indicated (one-way ANOVA & Bonferroni). **e** Addition of Nle3-VF or L3-VF to TAMRA-oAβ42 results in increased Aβ42 phagocytosis by cultured murine BV2 microglia. Phagocytosis of TAMRA-oAβ42 alone after incubation for 2 h ("TAMRA-oAβ42 (2 h)") versus TAMRA-oAβ42 after addition of Nle3-VF or L3-VF and co-incubation for 2 h ("(TAMRA-oAβ42 + Nle3-VF(or L3-VF)) (2 h)") was determined. Left panel, representative microscopic images of cells after incubation (6 h, 37°C) with the peptide solutions (prepared as in **d**) as indicated; red dots indicate TAMRA-Aβ42; scale bars, 100 µm. Right panel, amounts of phagocytic cells (% of total). Data are means ± SD from 20 ("TAMRA-oAβ42 (2 h)"), 12 ("(TAMRA-oAβ42 + Nle3-VF) (2 h)"), and 8 ("(TAMRA-oAβ42 + L3-VF)) (2 h)") biologically independent samples analyzed in 3 independent cell assays, each assay well analyzed in 3 fields of view; **P<0.01 (P=0.0098) for "(TAMRA-oAβ42 + L3-VF) (2 h)" versus TAMRA-oAβ42 and ****P<0.0001 for "(TAMRA-oAβ42 + Nle3-VF) (2 h)" versus TAMRA-oAβ42 as indicated (one-way ANOVA & Bonferroni).

## References included in Supplementary Information


1 Yan, L. M. *et al.* Selectively N-Methylated Soluble IAPP Mimics as Potent IAPP Receptor Agonists and Nanomolar Inhibitors of Cytotoxic Self-Assembly of Both IAPP and Abeta40. *Angew Chem Int Ed Engl* **52** 10378-10383, (2013).

2 Yan, L. M., Velkova, A., Tatarek-Nossol, M., Andreetto, E. & Kapurniotu, A. IAPP mimic blocks Abeta cytotoxic self-assembly: cross-suppression of amyloid toxicity of Abeta and IAPP suggests a molecular link between Alzheimer's disease and type II diabetes. *Angew Chem Int Ed Engl* **46**, 1246-1252, (2007).

3 Andreetto, E. *et al.* Identification of hot regions of the Abeta-IAPP interaction interface as high-affinity binding sites in both cross- and self-association. *Angew Chem Int Ed Engl* **49**, 3081-3085, (2010).

4 Franko, A. *et al.* Epigallocatechin gallate (EGCG) reduces the intensity of pancreatic amyloid fibrils in human islet amyloid polypeptide (hIAPP) transgenic mice. *Sci Rep* **8**, 1116, (2018).

5 Klunk, W. E., Jacob, R. F. & Mason, R. P. Quantifying amyloid by congo red spectral shift assay. *Methods Enzymol* **309**, 285-305, (1999).
