# Supplementary material for: Designed peptides as nanomolar cross-amyloid inhibitors acting via supramolecular nanofiber co-assembly
Source: Nat Commun. 2022 Aug 25;13:5004. doi: 10.1038/s41467-022-32688-0 (PMC9411207; doi:10.1038/s41467-022-32688-0)
Supplement: Supplementary file 3 — Description of additional supplementary files [file 41467_2022_32688_MOESM3_ESM.pdf]

Description Of Additional Supplementary Files

Supplementary Movie 1: 3D reconstruction of z-stacks of TAMRA-IAPP/Atto647N-Nle3-VF nanofiber bundle shown in Fig. 3d(middle panel); still image in Supplementary Figure 11d.

Supplementary Movie 2: 3D reconstruction of z-stacks of TAMRA-IAPP/Fluos-Nle3-VF nanofiber bundle shown in Fig. 3d(right panel); still image in Supplementary Figure 11e.

Supplementary Movie 3: 3D reconstruction of z-stacks of Atto647N-Nle3-VF-coated TAMRAfIAPP nanofiber bundle; still image shown in Supplementary Fig. 13e.

Supplementary Movie 4: 3D reconstruction of z-stacks of the supramolecular TAMRA-IAPP/FluosNle3-VF nanofiber co-assembly shown in Fig. 4c.

Supplementary Movie 5: 3D reconstruction of z-stacks of TAMRA-Aβ42/Fluos-Nle3-VF coassembly shown in Fig. 6e.

Supplementary Movie 6: 3D reconstruction of z-stacks of HiLyte647-Aβ42/TAMRA-fIAPP coassembly shown in Fig. 7g.

Supplementary Movie 7: 3D reconstruction of z-stacks of HiLyte647-Aβ42/TAMRA-fIAPP coassembly shown in Fig. 7h.

Supplementary Movie 8: 3D reconstruction of z-stacks of HiLyte647-Aβ42/Fluos-Nle3- VF/TAMRA-fIAPP co-assemblies shown in Fig. 7i/upper panel; still image in Fig. 7j.

Supplementary Movie 9: 3D reconstruction of z-stacks of HiLyte647-Aβ42/Fluos-Nle3- VF/TAMRA-fIAPP co-assembly shown in Fig. 7i/lower panel.
