# Supplementary material for: Designed peptides as nanomolar cross-amyloid inhibitors acting via supramolecular nanofiber co-assembly
Source: Nat Commun. 2022 Aug 25;13:5004. doi: 10.1038/s41467-022-32688-0 (PMC9411207; doi:10.1038/s41467-022-32688-0)
Supplement: Supplementary file 13 — Reporting Summary [file 41467_2022_32688_MOESM13_ESM.pdf]

nature portfolio

Corresponding author(s): Aphrodite Kapurniotu

Last updated by author(s): Jul 24, 2022

Reporting Summary

Nature Portfolio wishes to improve the reproducibility of the work that we publish. This form provides structure for consistency and transparency in reporting. For further information on Nature Portfolio policies, see our Editorial Policies and the Editorial Policy Checklist.

Statistics

For all statistical analyses, confirm that the following items are present in the figure legend, table legend, main text, or Methods section.

n/a	Confirmed	
☐	☒	The exact sample size (*n*) for each experimental group/condition, given as a discrete number and unit of measurement
☐	☒	A statement on whether measurements were taken from distinct samples or whether the same sample was measured repeatedly
☐	☒	The statistical test(s) used AND whether they are one- or two-sided<br>*Only common tests should be described solely by name; describe more complex techniques in the Methods section.*
☒	☐	A description of all covariates tested
☐	☒	A description of any assumptions or corrections, such as tests of normality and adjustment for multiple comparisons
☐	☒	A full description of the statistical parameters including central tendency (e.g. means) or other basic estimates (e.g. regression coefficient) AND variation (e.g. standard deviation) or associated estimates of uncertainty (e.g. confidence intervals)
☐	☒	For null hypothesis testing, the test statistic (e.g. *F*, *t*, *r*) with confidence intervals, effect sizes, degrees of freedom and *P* value noted<br>*Give P values as exact values whenever suitable.*
☒	☐	For Bayesian analysis, information on the choice of priors and Markov chain Monte Carlo settings
☒	☐	For hierarchical and complex designs, identification of the appropriate level for tests and full reporting of outcomes
☒	☐	Estimates of effect sizes (e.g. Cohen's *d*, Pearson's *r*), indicating how they were calculated

Our web collection on statistics for biologists contains articles on many of the points above.

Software and code

Policy information about availability of computer code

Data collection

ThT binding/MTT reduction: Instrument - VictorX3 Multilabel Reader (PerkinElmer), Software - Perkin Elmer 2030 Manager (V4.0)
Circular dichroism (CD): Instrument - Jasco 715 spectropolarimeter, Software - Spectra Manager V1.55.00 (Build 2)
Size exclusion chromatography: Instrument - Ultimate 3000 (Thermo Scientific) - Software Chromeleon (v7)
CR spectral shift assay: Clariostar Plus MTP reader (BMG Labtech) - Software - Clariostar Software (v. 5.70 R3)
Fluorescence spectroscopy: Instrument - Jasco FP-6500 fluorescence spectrophotometer, Software - Spectra Manager V1.54.03 (Build 1)
Transmission electron microscopy: Instrument - JEOL 1400 Plus electron microscope, Software - TEM Center (v. 1.7.19.2439)
X-ray diffraction: Instrument - Bruker D8 Venture diffractometer, Software - APEX 3 (v2019-1.0)
CLSM/STED: Instrument - Leica SP8 STED 3X microscope, Software - Leica Application Suite X (LAS-X) software package (v1.2)
2PM/FLIM-FRET: Instrument - Leica TCS SP8 DIVE two(multi)-photon microscope, Software - Leica Application Suite X (LAS-X) software package (v. 3.5.7.23225). In addition, for FLIM/FRET: Leica SP8 FLIM-FRET module capturing up to 1000 photons/pixel via TCSPC model and Leica's FALCON software (included in LAS-X software package; see above)
Cell uptake: Instrument - Leica DMi8 fluorescence microscope, Software - Leica Application Suite X (LAS-X) software package (v. 3.7.4.23463)
WB/Dot blots: Instrument - LAS-4000mini (Fujifilm) , Software - Image Reader LAS-4000 mini (V2.0)
LTP recordings: PC with software WinLTP (v.2.20) (WinLTP Ltd. and The University of Bristol, UK)
ESI-IMS-MS: Instrument - Synapt XS HDMS mass spectrometer (Waters), Software - MassLynx (v4.2)

Data analysis	OriginPro 2016G & Origin 2021; GraphPad Prism 5.0, 6.0, or 7.0; Microsoft Excel; Leica Application Suite X Core (v3.4.2), Leica LAS-X software package (v1.2), Leica LAS-X software package (v. 3.5.7.23225), deconvolutions: Huygens Professional (v.19.10) or Leica's LIGHTNING application (part of Leica's LAS-X software package), Leica's FALCON software (part of Leica's LAS-X software package); Image J (1.50i); MARS Data Analysis Software (V4.01 R2); Multi Gauge V3.1; GraFit (v.5); Adobe Photoshop Elements 7.0; APEX 3 (v2019-1.0); Image Reader LAS-4000 mini (V2.0); MassLynx (v4.2); WinLTP Software (v.2.20); Perkin Elmer 2030 Manager (V4.0); Spectra Manager V1.55.00 (Build 2); Spectra Manager V1.54.03 (Build 1); Chromeleon (v7)

For manuscripts utilizing custom algorithms or software that are central to the research but not yet described in published literature, software must be made available to editors and reviewers. We strongly encourage code deposition in a community repository (e.g. GitHub). See the Nature Portfolio guidelines for submitting code & software for further information.

Data

Policy information about availability of data

All manuscripts must include a data availability statement. This statement should provide the following information, where applicable:

- Accession codes, unique identifiers, or web links for publicly available datasets
- A description of any restrictions on data availability
- For clinical datasets or third party data, please ensure that the statement adheres to our policy

The authors declare that the data supporting the findings of this study are available within the paper and its Supplementary Information files. Source data are provided with this paper. A.K., J.B., and K.T. are co-inventors of the European patent application 22 158 021.0 (applicant Technical University of Munich) (status: pending) related to ACMs, their hetero-assemblies, and potential biomedical applications.

Field-specific reporting

Please select the one below that is the best fit for your research. If you are not sure, read the appropriate sections before making your selection.

☒ Life sciences ☐ Behavioural & social sciences ☐ Ecological, evolutionary & environmental sciences

For a reference copy of the document with all sections, see nature.com/documents/nr-reporting-summary-flat.pdf

Life sciences study design

All studies must disclose on these points even when the disclosure is negative.

Sample size	Sample size calculations were not performed. Sample size for the biochemical assays (e.g. ThT binding, MTT reduction assays, fluorescence spectroscopic titrations) was typically n=3 (biologically independent samples) which is the standard sample number in biochemical experiments and consistent with previous work (Spanopoulou et al. Angew. Chem. Int. Ed. (2018); Andreetto et al. Angew. Chem. Int. Ed. (2015); Yan et al. Angew. Chem. Int. Ed. (2007)). In the case of phagocytosis assays, sample sizes were based on similar studies in the field and on magnitude and concistency of the differences between various treatments in our own experiments (Hu et al. PNAS (2009), Chung et al. JBC (1999); Aftabizadeh et al., ACS Chem Neurosci. (2019); Kontos et al. NCOMMS (2020)). For ex vivo LTP measurements, sample size ranged typically between 6 and 12 based on our experience with previous studies (Spanopoulou et al. Angew. Chem. Int. Ed. (2018); Andreetto et al. Angew. Chem. Int. Ed. (2015); Yan et al. Angew. Chem. Int. Ed. (2013)).
Data exclusions	Data were excluded only in rare instances of principal technical issues.
Replication	The number of independent experiments and samples underlying the data shown in graphs and tables is stated in the legends. For instance, ThT assays/MTT assays/fluorescence spectroscopic titrations were usually repeated at least 3 times if not stated otherwise. CD spectra are means of 3 consecutive measurements from the same sample. Results from SEC/ANS/cross-linking assays/pulldowns/dot blots/ESI-IMS-MS are representative of at least 2 independent experiments if not stated otherwise. TEM/CLSM/STED/2PM images and related data are from peptide samples analysed in various different fields of view and represent images obtained in different fields of view and typically in at least two similar sample preparations. FLIM-FRET analyses were done on representative hetero-assemblies. Phagocytosis assays were typically performed using at least 4 independent samples of the same peptide and LTP measurements were done on at least 6 mouse hippocampal slices per peptide sample. Overall, results were reproducible when established protocols were applied.
Randomization	In general, randomization of individual peptide samples studied by the in vitro biophysical and biochemical experiments (e.g. ThT, MTT reduction assays, SEC, fluorescence binding assays, CD spectroscopy, 2PM, FLIM-FRET, dot blots etc.) was not performed as this is experimentally not an option. Standard protocols for sample preparations and treatments were applied as described in the Methods section. However, the cells used in all cellular assays (i.e. MTT reduction and phagocytosis) were randomly plated in the MTP. Also, in some cases, i.e. quantification of peptide uptake via cellular phagocytosis assays or STED-/TEM-based determination of fibril dimensions, the various different fields of view were randomly selected for the data analysis. Furthermore, the ex vivo LTP measurements were performed with randomly selected hippocampal slices.
Blinding	Since the investigators who performed the experiments in most biochemical/biophysical experiments also analysed the data, blinding was not possible. However, data aquisition and analysis was performed using standard protocols in an unbiased manner with pre-determined conditions and criteria. Indeed, blinding is usually not used in biophysical/biochemical experiments. Of note, same results were obtained when some of the experiments (e.g. by ThT binding, MTT reduction assay, cross-linking assays, 2PM, TEM) were performed by different investigators who applied same standard protocols. Also, analysis of CLSM, STED, & 2PM data and FLIM-FRET studies were always performed by 2 investigators together (with one of them not having been involved in sample preparation).

Reporting for specific materials, systems and methods

We require information from authors about some types of materials, experimental systems and methods used in many studies. Here, indicate whether each material, system or method listed is relevant to your study. If you are not sure if a list item applies to your research, read the appropriate section before selecting a response.

Materials & experimental systems

n/a	Involved in the study
☐	☒ Antibodies
☐	☒ Eukaryotic cell lines
☒	☐ Palaeontology and archaeology
☐	☒ Animals and other organisms
☒	☐ Human research participants
☒	☐ Clinical data
☒	☐ Dual use research of concern

Methods

n/a	Involved in the study
☒	☐ ChIP-seq
☒	☐ Flow cytometry
☒	☐ MRI-based neuroimaging

Antibodies

Antibodies used

Anti-rabbit IgG (whole molecule), gold conjugate 10 nm, produced in goat; Sigma-Aldrich, G-3779-.4ML
Anti-mouse IgG (whole molecule), gold conjugate 5 nm, produced in goat; Sigma-Aldrich, G7527-.4ML
Rabbit anti-Amylin (Human) IgG, produced in rabbit; Peninsula Laboratories/BMA Biomedicals, T-4149
(abbreviation in Methods part: rabbit polyclonal anti-IAPP antibody)
Anti-IAPP mouse monoclonal antibody, (IAPP fibril-specific), Clone 91E7; Synaptic Systems (abbreviation in Methods part: fIAPP-specific mouse anti-fIAPP antibody) (not yet part of the catalogue; validation e.g. by Franko A. et al. Sci. Rep. 8, 1116 (2018).
IAPP-specific mouse anti-fIAPP antibody23 (Synaptic Systems; Cl. 91E7, 1:500) (the provider does not have a catalogue number for this product)
(Anti-)oligomer A11 polyclonal antibody (Thermo Fischer Scientific), AHB0052
Anti-β-Amyloid Protein (1-40) antibody produced in rabbit; Sigma-Aldrich, A8326-.5ML
Anti-amyloid beta [6E10], IgG1, Mouse, Monoclonal; Biozol, ABA-AB00714-1.1-BT
Anti-Mouse IgG H&L, Secondary Antibody, produced in goat; Abcam, ab6789 (abbreviation in Methods part: goat anti-mouse-POD)
ECL Anti-Rabbit IgG, horseradish peroxidase-linked species-specific whole antibody (from donkey) (25005179), Secondary Antibody; Thermo Fisher Scientific, 10794347 (Amersham code NA934) (abbreviation in Methods part: donkey anti-rabbit POD)

Validation

Anti-IAPP (fibril-specific), from mouse, Clone 91E7: Franko, A. et al., Sci Rep 8, 1116 (2018) (binding to fIAPP confirmed also by our studies (e.g. Fig. 4g & Supplementary Fig. 9a)).
All other antibodies were well known in the field and commercially available antibodies which are used for the recommended application as stated by the supplier.
Anti-rabbit IgG (whole molecule), gold conjugate 10 nm, produced in goat: https://www.sigmaaldrich.com/DE/de/product/sigma/g3779
Anti-mouse IgG (whole molecule), gold conjugate 5 nm, produced in goat: https://www.sigmaaldrich.com/DE/de/product/sigma/g7527
Rabbit Anti-Amylin (Human) IgG: http://www.bma.ch/files/product/t-4149-amylin-igg-lot-a18po2147_1.pdf
Anti-β-Amyloid Protein (1-40) antibody produced in rabbit: https://www.sigmaaldrich.com/DE/de/product/sigma/a8326
Anti-amyloid beta [6E10], IgG1, Mouse, Monoclonal: https://www.biozol.de/de/product?q=ABA-AB00714-1.1-BT
(Anti-)oligomer A11 polyclonal antibody (Thermo Fischer Scientific): https://www.thermofisher.com/antibody/product/Oligomer-A11-Antibody-Polyclonal/AHB0052

Eukaryotic cell lines

Policy information about cell lines

Cell line source(s)

PC12 cells were obtained from DSMZ - German Collection of Microorganisms and Cell Cultures GmbH (DSMZ no. ACC 159). RIN5fm cells were obtained from T.E. Rucinsky at the Washington University Tissue Culture Support Center. BV2 microglia (RRID:CVCL_0182) were obtained from Dr. M. Kipp (Rostock University) who had purchased them from ATCC (EOC2 (CRL-2467)); original reference: Blasi lab (Blasi E, Barluzzi R, Bocchini V, Mazzolla R, Bistoni F (1990) Immortalization of murine microglial cells by a v-raf/v-myc carrying retrovirus. J Neuroimmunol 27:229–237.

Authentication

Cell lines were not authenticated by us. Authentication of PC12 and RIN5fm was performed by the providers. BV2 microglia cells (RRID:CVCL_0182) were initially obtained from Dr. M. Kipp (Rostock University) who had purchased them from ATCC (EOC2 (CRL-2467)); the clone was sub-cultured and not used beyond passage 20. The cell line was characterized in the original publication by Blasi et al (1990) and more recently and comprehensively in Henn et al, published in Altex 26, 2/09, entitled "The Suitability of BV2 Cells as Alternative Model System for Primary Microglia Cultures or for Animal Experiments Examining Brain Inflammation" (2009), showing that these cells display many characteristics typical of primary murine microglia.

Mycoplasma contamination

Primary cells (BMDMs) were freshly obtained from BL6/J mice and used as described in the Methods section. They were

Mycoplasma contamination	handled in a Mycoplasma-free reserved hood. Cell lines were tested mycoplasma-free by the provider. They were used without being further tested.
Commonly misidentified lines (See ICLAC register)	-none-

Animals and other organisms

Policy information about studies involving animals; ARRIVE guidelines recommended for reporting animal research

Laboratory animals	For ex-vivo LTP assays: C57BL/6N mice, male, 6-8 weeks (Charles River). Animal housing conditions: max. 6 animals per cage; food and water ad libitum, 12h dark/light cycle, climate controlled rooms with 23±0.5°C and humidity 55±10%. Animal experiments were approved by the local authorities (animal ethics approval 4-016-18) and were performed according to the German animal protection law.<br>For phagocytosis assays: for isolation of BMDMs, C57BL6/J mice (8-16 weeks of age, both sexes (Charles River)) were used. Mice were housed and bred under standardized (12h dark/light cycle, climate controlled rooms with 21±1°C and humidity 55%) and specific pathogen-free conditions in the animal facility of the Center for Stroke and Dementia Research (CSD) in Munich, with free access to food and water. Animal experiments were approved by the local authorities (animal ethics approval ROB-55.2-2532.Vet_02-18-040 / KSP-M) and were performed according to the German animal protection law.
Wild animals	Study did not involve wild animals.
Field-collected samples	Study did not involve samples collected from the field.
Ethics oversight	Ethical committee on animal care and use of the government of Bavaria (Regierung von Oberbayern, ROB), Germany.

Note that full information on the approval of the study protocol must also be provided in the manuscript.
